# Single nucleotide variation catalog from clinical isolates mapped on tertiary and quaternary structures of ESX-1-related proteins reveals critical regions as putative Mtb therapeutic targets

**DOI:** 10.1128/spectrum.03816-23

**Published:** 2024-06-14

**Authors:** Oren Tzfadia, Abril Gijsbers, Alexandra Vujkovic, Jihad Snobre, Roger Vargas, Klaas Dewaele, Conor J. Meehan, Maha Farhat, Sneha Hakke, Peter J. Peters, Bouke C. de Jong, Axel Siroy, Raimond B. G. Ravelli

**Affiliations:** 1 Mycobacteriology Unit, Institute of Tropical Medicine, Antwerp, Belgium; 2 Departamento de Bioquímica, Facultad de Medicina, Universidad Nacional Autónoma de México, Mexico City, Mexico; 3 Clinical Virology Unit, Institute of Tropical Medicine, Antwerp, Belgium; 4 ADReM Data Lab, University of Antwerp, Antwerp, Belgium; 5 Department of Biomedical Informatics, Harvard Medical School, Boston, Massachusetts, USA; 6 Department of Biosciences, Nottingham Trent University, Nottingham, United Kingdom; 7 Division of Nanoscopy, Maastricht Multimodal Imaging Institute (M4i), Maastricht University, Maastricht, the Netherlands; 8 Unité de soutien à l'Institut Européen de Chimie et Biologie (IECB), CNRS, INSERM, IECB, US1, Université de Bordeaux, Pessac, France; CNRS - University of Toulouse, Toulouse, France

**Keywords:** SNV, virulence factors, *Mycobacterium tuberculosis*, protein structure-function, AlphaFold

## Abstract

Proteins encoded by the ESX-1 genes of interest are essential for full virulence in all *Mycobacterium tuberculosis* complex (Mtbc) lineages, the pathogens causing the highest mortality worldwide. Identifying critical regions in these ESX-1-related proteins could provide preventive or therapeutic targets for Mtb infection, the game changer needed for tuberculosis control. We analyzed a compendium of whole genome sequences of clinical Mtb isolates from all lineages from >32,000 patients and identified single nucleotide polymorphisms. When mutations corresponding to all non-synonymous single nucleotide polymorphisms were mapped on structural models of the ESX-1 proteins, fully conserved regions emerged. Some could be assigned to known quaternary structures, whereas others could be predicted to be involved in yet-to-be-discovered interactions. Some mutants had clonally expanded (found in >1% of the isolates); these mutants were mostly located at the surface of globular domains, remote from known intra- and inter-molecular protein–protein interactions. Fully conserved intrinsically disordered regions of proteins were found, suggesting that these regions are crucial for the pathogenicity of the Mtbc. Altogether, our findings highlight fully conserved regions of proteins as attractive vaccine antigens and drug targets to control Mtb virulence. Extending this approach to the whole Mtb genome as well as other microorganisms will enhance vaccine development for various pathogens.

**IMPORTANCE:**

We mapped all non-synonymous single nucleotide polymorphisms onto each of the experimental and predicted ESX-1 proteins’ structural models and inspected their placement. Varying sizes of conserved regions were found. Next, we analyzed predicted intrinsically disordered regions within our set of proteins, finding two putative long stretches that are fully conserved, and discussed their potential essential role in immunological recognition. Combined, our findings highlight new targets for interfering with *Mycobacterium tuberculosis* complex virulence.

## INTRODUCTION

*Mycobacterium tuberculosis* causes tuberculosis (TB) in humans and other mammals. This remarkably monomorphic pathogen shares 99.9% nucleotide similarity and identical 16S rRNA in its larger *Mycobacterium tuberculosis* complex (Mtbc), unlike the diversity seen in other bacteria (1–4). In the past decades, extensive research has been done to clarify the precise virulence mechanisms of the Mtbc. The protein ESAT-6 (6 kDa early secretory antigenic target, also known as EsxA) was identified as its main secreted virulence factor (5, 6), associated with the protein EsxB. The secreted heterodimer is critical for Mtbc virulence through its cytolytic activity (7–10). Attenuation of the BCG (*Mycobacterium bovis* strain Calmette Guérin) was achieved by the spontaneous loss of the chromosomal region RD1 (region of difference 1) that carries the gene *esxA* (11). Likewise, BCG strains have acquired further attenuation mutations in addition to the loss of RD1, as indicated by the finding that complementation of BCG with the extended RD1 region can increase the virulence of the recombinant strain to some extent but not to the full level of a typical *M. bovis* cattle isolate. Similarly, deletion of RD1 in Mtb caused decreased virulence similar to that of BCG *in vitro* (12). Despite the spontaneous loss of an overlapping section of the RD1 region, *Mycobacterium microti* can show different degrees of virulence in animal models. Some *M. microti* strains, which were previously used as vaccines, were found to be highly attenuated, whereas some other strains show higher virulence (13).

The RD1 region contains genes of the ESAT-6 secretion system 1, ESX-1 (11, 14). ESX-1 is a member of the type VII secretion systems (T7SS) family and is essential for full virulence in all Mtbc lineages (L1–L8) as well as in the closely related pathogenic *Mycobacterium marinum*, *Mycobacterium kansasii* (15), and *Mycobacterium leprae* (16). The ESX-1 genes of interest (GOI) are mainly located within the extended RD1 locus but also include multiple genes across different loci and are required for the building, functioning, and regulation of ESX-1, the transport of virulence factors, and their membrane lysis activity. The ESX-1 GOI encodes 31 proteins (ESX-1-related proteins, see Table S14) that can be divided into four functional categories: 7 substrates (products that are secreted during virulence), 6 inner-membrane core components (proteins part of the secretion machinery), 8 regulators (transcription factors), and 10 peripherals (exact function yet to be determined); the mechanism by which the substrates translocate through the mycobacterial outer membrane has not been solved yet, and the proteins potentially involved in that step have not been identified.

The structure of the ESX-1 inner core complex has not been solved yet; however, those of ESX-3 (17) and ESX-5 (18) have been deciphered, and atomic models for a few ESX proteins (in isolation or as part of a complex) have also been experimentally solved. Accurate structure prediction of single proteins has become available through artificial intelligence (AI) techniques (19, 20), and the obtained models generally compare well to the experimental ones, thus allowing for large structural bioinformatics screens *in silico*. Using AI-generated protein models also allows for the building of higher-order protein complexes together with homologous templates such as the ESX-3 and ESX-5 core machineries in the case of ESX-1, thus allowing for the exploration of the inter-molecular interfaces within.

Despite their high genomic similarity, Mtbc lineages (L) differ significantly in the host immune response they elicit, host tropism, phenotypes, drug resistance, and transmissibility (21–24). So far, most research on the ESX-1 machinery has focused on the Mtbc L2 and L4 because of their widespread geographic range and the availability of laboratory-adapted reference strains such as H37Rv (L4) and HN878 (L2). Thus, our knowledge on the genomic differences and convergence in the ESX-1 genes across the Mtbc is limited.

To obtain a deeper understanding of virulence across Mtbc, we generated single nucleotide polymorphism (SNP) catalog found in the ESX-1 GOI for Mtbc lineages (L1–L6) of human importance. We used whole-genome sequencing (WGS) data from >32,000 publicly available clinical isolates to shed light on the intricate landscape of virulence proteins. We investigated—with advanced bioinformatics methods—the spatial distribution of these genetic polymorphisms at the protein level from known essential amino-acid positions to protein-protein interfaces. In the process, we identified new hyperconserved protein domains in the peripherals and substrates of ESX-1: those coincide with intrinsically disordered regions, thus highlighting their essential nature in the intracellular survival of Mtb.

## RESULTS

First, we provide evidence illustrating the variation tolerance of the ESX-1 GOI, confirming that it is the most non-synonymous single nucleotide polymorphism (nSNP)-dense group of genes within the Mtb genome (25). Next, we identified several genomic regions, including variants that arose independently under positive selection as done by Vargas et al. (25). We then examined the amino-acid locations that bear abundant SNP counts. In 31 ESX-1 genes, only 21 nSNPs were found in more than 1% of the isolates; given their extensive human-to-human passage, we considered them fully functional. None of these nSNPs resulted from convergence but were due to either opportunistic sampling of the data set or occurring in ancestral lineages. The data also revealed which parts of the 31 proteins are fully conserved. We mapped all nSNPs onto each of the experimental and predicted ESX-1 proteins’ structural models and inspected their placement. Varying sizes of conserved regions were found, and some proteins showed clear polarity in their nSNP distribution. We then scrutinized some essential motifs [such as Walker motifs (26), secretion signals, SS-bonds, and post-translational modification sites] and identified less than 0.01% of isolates bearing nSNPs in those locations. Next, we analyzed predicted intrinsically disordered regions (IDRs) within our set of proteins, finding two putative long stretches that are fully conserved, and discussed their potential essential role in immunological recognition. Finally, we compared known and predicted quaternary structures, correlated interaction interfaces with nSNP distribution maps, and experimentally validated one unexpected mutation on the interaction interface of EsxA and EsxB, which still permits complex formation. Combined, our findings highlight new targets for interfering with Mtbc virulence.

### Consolidating SNPs for ESX-1 GOI

**Fig 1:**
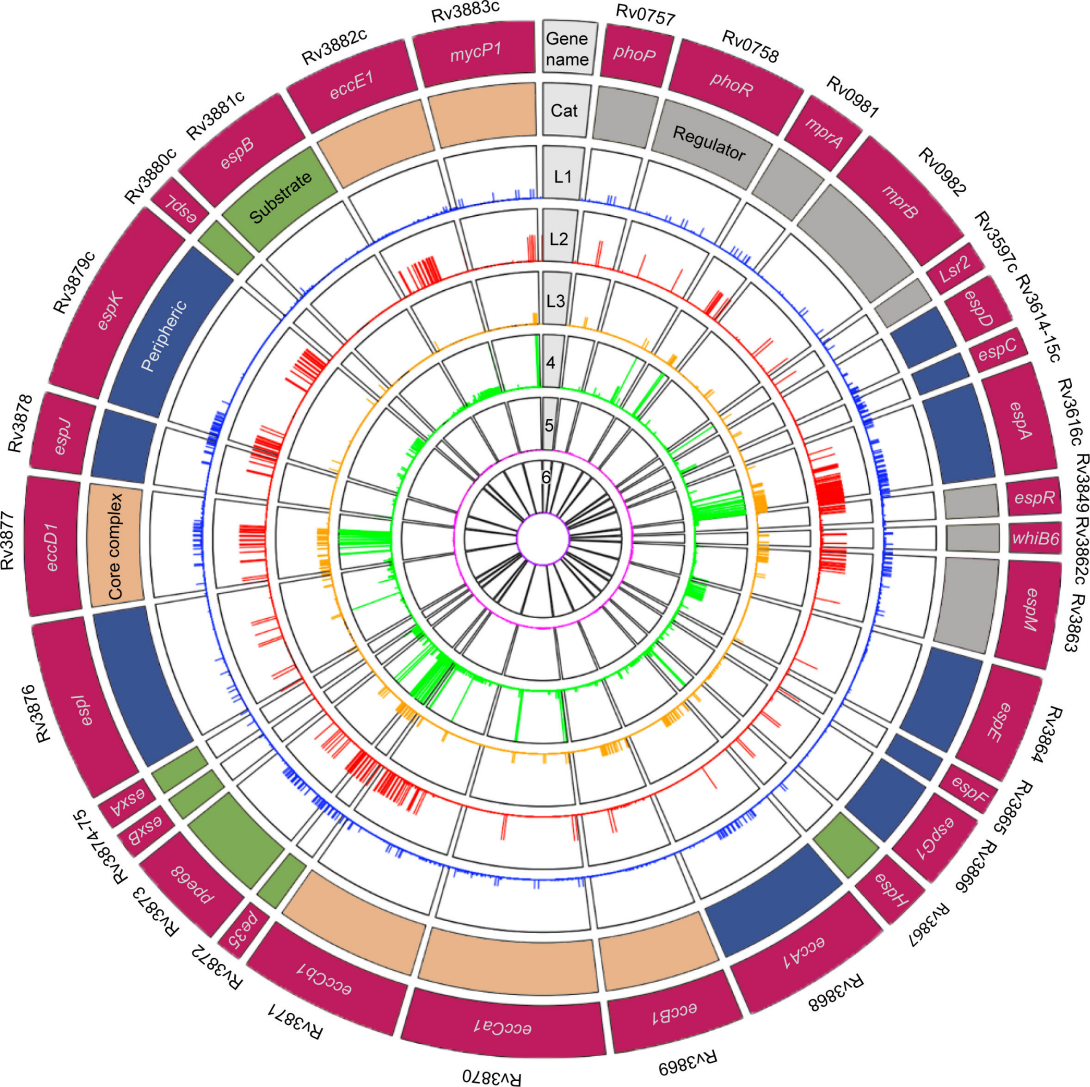
nSNP catalog for 31 ESX-1 GOI and per MTBc lineage. Circos plot visualization of distinctive nSNPs counted in the data was made from merged variant files (MTBseq pipeline) and then filtered by uniqueness per lineage (not shared with other lineages). The outer lane depicts gene names. The first lane is color coded by functionality: green for substrates, gray for regulators, salmon for core complex, and blue for peripheral. The next six inner lanes designate lineage stratification (L1–L6).

A collection of 32,399 unique Mtbc isolates, including clinical Mtbc isolates (25) L1–L6 (NCBI), L7 (27), as well as *M. bovis* (B.C. de Jong, unpublished data), was collated (Fig. 1; Fig. S1). The presence of an ESX-1 machinery and its substrates, essential to Mtb virulence, in virulent clinical isolates from human TB patients may be considered as positively selected strains for virulence in a genetic selection screen. For the ESX-1 GOI, we examined 31 genes encoding 31 proteins with a total of 11,167 amino acids, of which 8,616 displayed an SNP: 2,742 synonymous mutation sites (sSNPs) and 5,874 nSNPs. More than half (52%) of the encoded amino acids had at least one nSNP. Figures S2–S4 visualize the nSNPs mapped on the AlphaFold2-predicted structures for each of the 31 ESX-1 GOI.

### Converging evolution

From convergence analysis, two silent sSNPs in *espI* in four independent lineages (L1, L4, L3, and L8) in a total of 10 unrelated clinical isolates were identified and confirmed by Sanger sequencing (Fig. S5; Table S2); these sSNPs featured a six cytosine repeat at codons 134–135. In cancer, this type of motif has been linked to changes in methylation (28). Long PacBio reads could provide information about whether these mutations affect the methylation state of ESX-1.

### Conservation on 3D level

We mapped all nSNPs onto each of the known or predicted 3D structural models and inspected their spatial distribution. Varying sizes of conserved regions were found, as shown by the dominant blue color of the predicted protein structures ( Fig. S2–S4). Some proteins showed clear polarity; for example, nSNPs of the regulators WhiB6, PhoR, PhoP, MprA, and MprB accumulated on one side of their surface, while the opposite side showed no nSNP (Fig. S3).

Comparing the prevalence of nSNPs across the 31 ESX-1 GOI, we found that WhiB6 (65.2%) and PhoR (54.2%), as well as two members of the ESX-1 inner membrane core complex EccB1 (54.4%) and EccE1 (53.8%), had the highest percentage of amino acid changes. This trend of clustered nSNPs to one position was also observed for *esxB* (96%), *PE35* (99%), *espA* (91%), *mprB* (94%), *mprA* (83%), *espH* (78%), *espE* (77%), and *espL* (69%) (Fig. S6–S9).

### Hotspots in ESX-1 GOI

**Fig 2:**
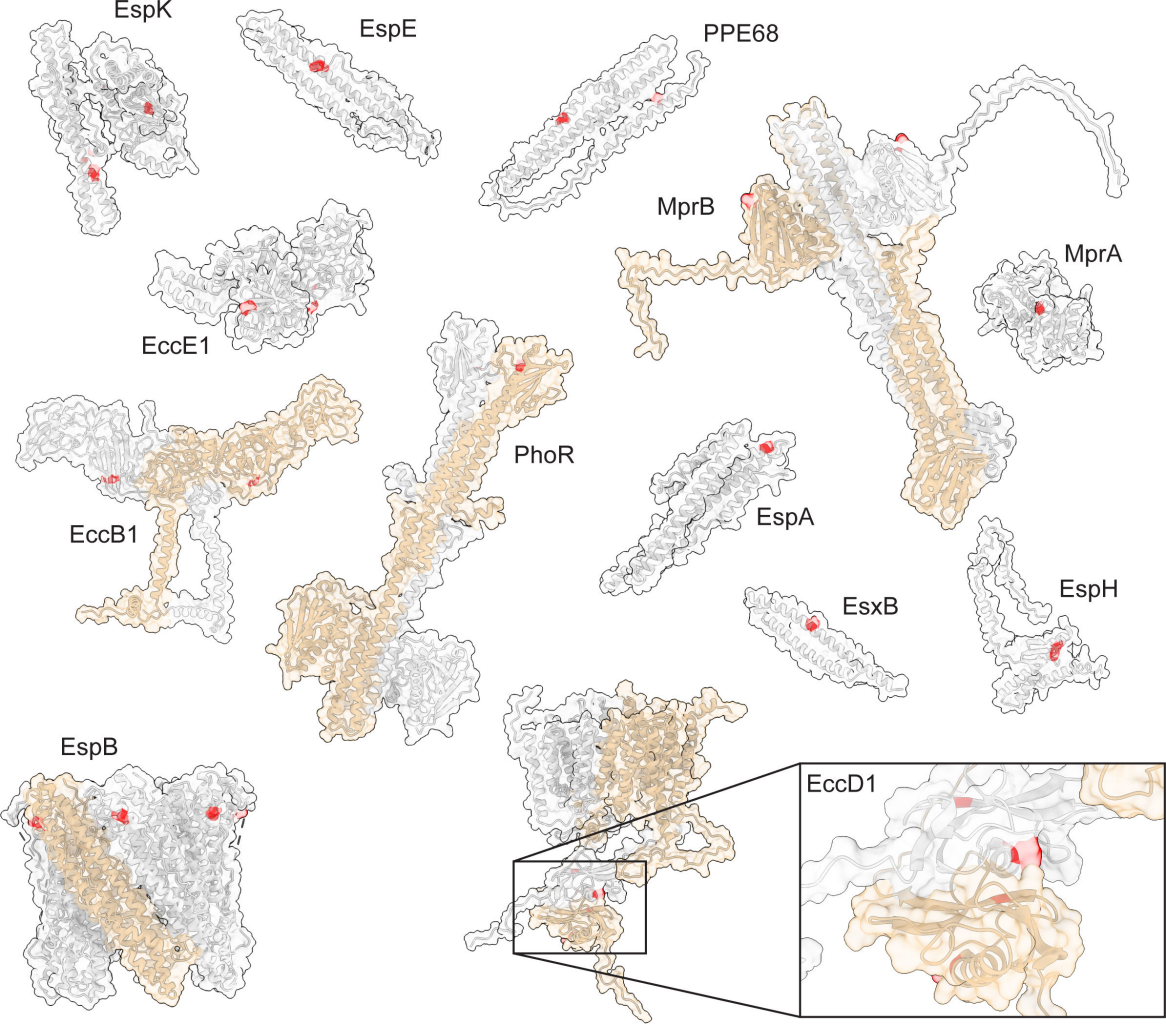
Spatial distribution of nSNPs hotspots in ESX-1 components. Hotspots (depicted in red) were mapped onto the structural models of the ESX-1 proteins that harbour them: the EspB heptamer was solved experimentally (N-terminal moiety, PDB: 7P13), EccB1 and EccD1 dimers were built by a combination of AlphaFold2 prediction and molecular threading using their homologs from ESX-3 and ESX-5 as templates, the PhoR and MprB dimers were predicted using AlphaFold2-multimer, the other models were gathered from the AlphaFold Protein Structure Database (https://alphafold.ebi.ac.uk/). Regions of proteins predicted not to be folded and not containing mutation hotspots are omitted from the models. A hotspot is classified as such when the same nSNPs is found in more than 300 clinical isolates (constituting over 1% of the isolates). Homo-oligomers are depicted with a one subunit in colour tan.

After excluding the two polymorphisms in *espI* due to convergent evolution, we identified 78% of the nSNPs in so-called hotspots [in which the same mutation was found in over 1% of the clinical isolates (*n* > 300)], reflecting a phylogenetically more “basal” polymorphism with clonal expansion. We identified 21 hotspots (Fig. 2) in 13 of the 31 ESX-1 GOI, involving all lineages (Table S3). Only four proteins showed more than one hotspot (PPE68, EccD1, EccE1, and EspK), while nine showed each a single hotspot (PhoR, MprA, MrpB, EspA, EspE, EccB1, EsxB, EspH, and EspB). The two most abundant nSNPs were excluded as hotspots (T192I in EspA and E99* in PE35) since they included almost the entire data set, suggesting those polymorphisms appeared in the H37Rv reference strain instead; the L339H MprB variant appeared in half the data set. Hotspots occurred in all protein categories: we identified them in three regulators, three peripherals, four substrates, and three core components. Upon mapping the hotspots onto the structural models, we found them predominantly located on the surface of the proteins, including both polar and apolar residues (Fig. 2). Visual inspection of the experimental as well as predicted protein structures of these 13 proteins and 21 hotspots showed very few polar and H-bond interactions of the hotspot side chains within the protein itself (data not shown).

**Fig 3:**
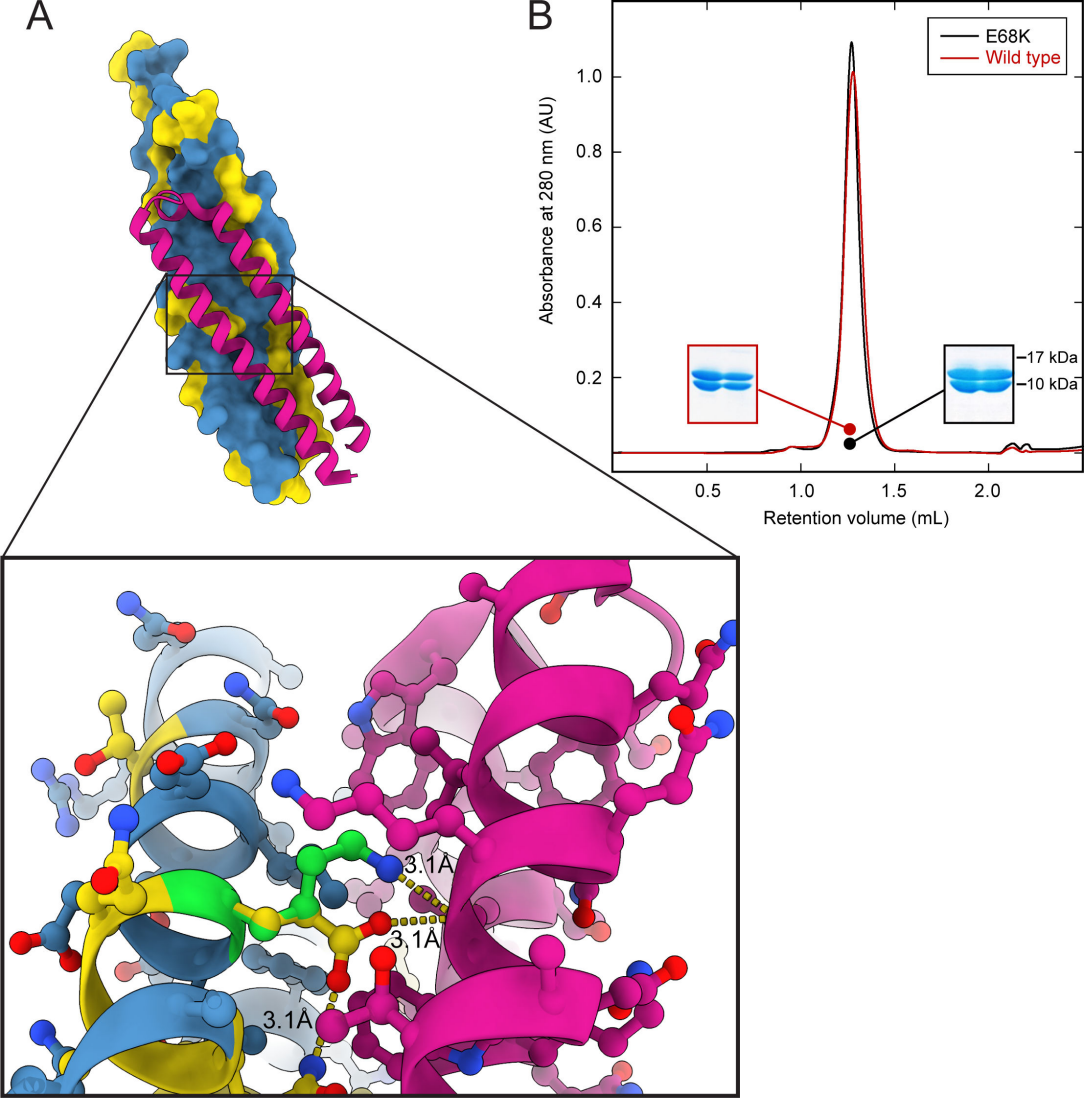
Hotspot E68K in EsxB does not affect the interaction with EsxA. (A) Structure depicts interaction between wild-type protein EsxA (magenta) and EsxB (blue = 0 SNPs, yellow > 0 SNPs). Zoom-in region of residue E68 showing intra- (with Q65 of EsxB) and intermolecular contacts (with the main chain of W58 of EsxA). Lysine replacing E68 is displayed in green with its respective interaction. (B) Size-exclusion chromatogram shows conserved interaction between EsxA and EsxB regardless of hotspot E68K (black curve); wild-type EsxB (red curve) is shown for comparison. Insets correspond to the respective fractions resolved in a SDS-PAGE, showing both proteins (EsxA 9.9 kDa, EspB 10.8 kDa).

Since the effectors EsxA and EsxB are known to assemble into a heterodimer prior to its secretion by the ESX-1 system, we investigated the most prevalent EsxB variant E68K as it involves a residue buried in the interface with EsxA (29, 30) and determined how it affected the assembly. We expressed and purified the mutant protein and showed an unharmed interaction with EsxA (Fig. 3), confirming earlier predictions by molecular modeling that the E68K mutation would have minimal impact on the interface between EsxB and EsxA.

### Essential motifs and binding interface regions

Next, we analyzed the presence of nSNPs in the two motifs known to be essential for secretion in the substrates and peripheral proteins: WxG and YxxxD/E. Overall, these secretion motifs were found to be highly conserved. Only nine nSNPs were found at the tryptophan and four at the glycine sites of the WxG motif, while for the YxxxD/E motif 13 and 2 nSNPs were identified at the tyrosine and aspartic acid sites, respectively.

Some of the ESX-1 core components contain ATPase domains, the hydrolysis of which powers the secretion of the ESX-1 substrates through the mycobacterial membranes. These domains require the presence of Walker motifs for the proper binding and hydrolysis of nucleotides. Thus, we analyzed whether nSNPs occurred in these motifs. The Walker A motif, also called the “P-loop” for its phosphate-binding loop, exhibits the classical pattern (G/A)xxxxGK(T/S) (31). Our data show a total number of six nSNPs within this motif for the total of three ATPase domains found in EccCa1 and EccCb1; no nSNP was found in the P-loop motif of EccA1 (Table S4). The Walker B motif (hhhhD, where h is any hydrophobic residue) is fully conserved in all three proteins. The peripheral protein EspI harbors another P-loop NTPase domain; its role in downregulating ESX-1-mediated secretion when Mtb cellular ATP levels are low has been established (32), and a binding site of moderate affinity to ATP has been identified within its Walker A motif (33). In EspI, this motif is well conserved with only six nSNPs, and the essential ATP-binding residue Lys425 is unaltered.

**Fig 4:**
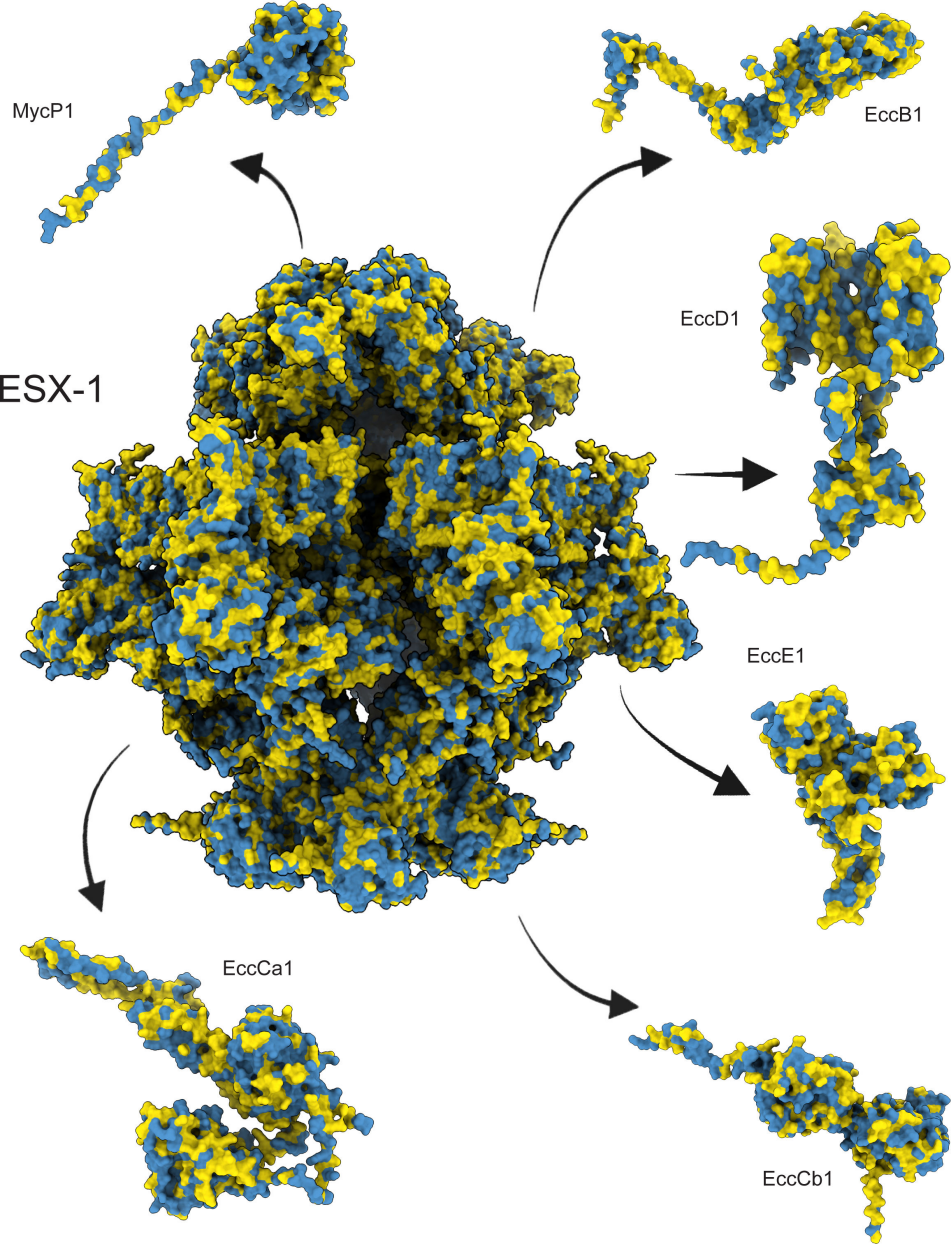
nSNPs mapped onto the AlphaFold2-predicted models of an ESX-1-assembled core complex modeled by molecular threading onto ESX-3 and ESX-5. The complex was modeled as assembled in the Mtbc cytoplasmic membrane, where one subunit of EccB1, EccCa1, EccCb1, and EccE1 and two of EccD1 form a protomer, two protomers are stabilized by a copy of MycP1, which then trimerizes to form the hexamer. Color code: blue, 0 nSNPs; yellow>0 nSNPs in>32,000 clinical isolates.

The core membrane component (e.g., the secretion machine) of ESX-1 is critical for the virulence of the bacteria and represents an important target for the development of new antitubercular agents. It is built from multiple copies of four ESX conserved components (Ecc), named EccB, EccC, EccD, and EccE, and is stabilized by the protease MycP1, resulting in a large ~1,500 kDa particle. Therefore, we looked for SNP trends in the ESX-1 inner membrane core complex. Because no experimental structure of this complex has been elucidated to date, we modeled it as a trimer of protomer dimers and mapped the identified nSNPs onto it. Our data set revealed a large overall number of mutations within each of its components: 5,923 in EccB1, 2,679 in EccCa1, 3,614 in EccCb1, 6,760 in EccD1, 7,722 in EccE1, and 1,148 in MycP1 (total of 27,846 in the five proteins combined). No obvious trend was found in terms of nSNP clustering or conservation (Fig. 2 and Fig. 4; Fig. S9)

Finally, we analyzed post-translational modification sites, allowing for glycosylation and phosphorylation, which were shown to play a significant role in Mtb adaptive processes. A recent study mapped glycoproteomic patterns of clinical isolates of Mtb (34). Consequently, we honed in on the 31 ESX-1 GOI, which contains 27 glycosylation sites in total. The majority of sites (18/27) did not harbor nSNPs; the other nine sites counted only 31 nSNPs in >32,000 isolates (0.01%) (Table S6). Moreover, complete conservation (0 nSNP) was observed in the phosphorylation sites for EspJ (Ser70) (35) and the transcription regulators PhoR (His259) (36) and MprB (His249) (37). Together, these data demonstrate that functional regions essential to ESX-1 function are largely conserved within the set of 31 ESX-1 GOI.

### Intrinsically disordered regions

**Fig 5:**
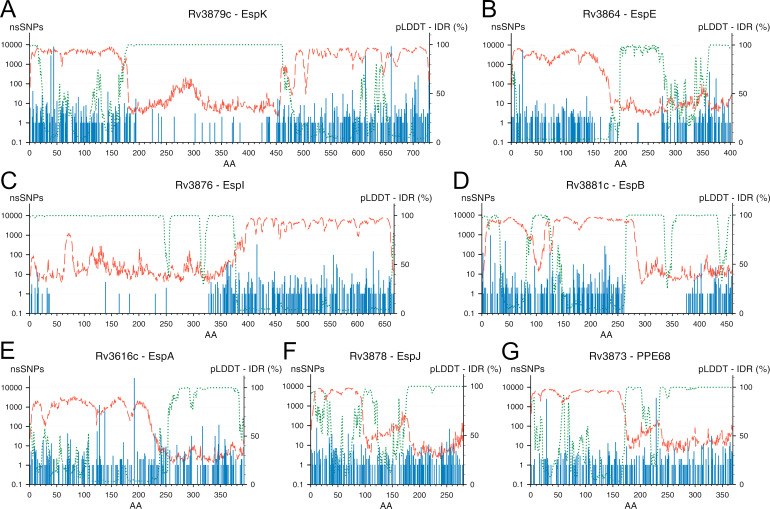
ESX-1 components with the largest predicted IDR domains. The amino-acid sequences of the ESX-1 GOI were analysed with the AlphaFold2 and ODiNPred algorithms. The proteins with IDRs were identified as the longest low-pLDDT, high-disorder scores. A highly conserved IDR was identified in EspK (**A**). EspE (**B**), EspI (**C**) and EspB (**D**) displayed both conserved and polymorphic IDR sections. Long polymorphic IDR regions were identified in EspA (**E**), EspJ (**F**) and PPE68 (**G**). Per amino-acid position (X axis): nSNPs counts (blue bars, left Y axis in logarithmic scale), AlphaFold2 confidence (red dashes as pLDDT score on left Y axis as percentage) and ODiNPred disorder scores (green dots as IDR score also on the right Y axis as percentage).

The predicted AlphaFold structural models contain regions of low and very low confidence scores (respectively, <70 and <50). The percentage of low-confidence regions varies for different species and is relatively small for bacterial proteomes such as *M. tuberculosis* (13.29%) (38). We analyzed whether these regions of low confidence in the proteins encoded by the ESX-1 GOI reflect intrinsically disordered regions (39), which tend to be hydrophilic unstructured protein structures thought to be disproportionately involved in interactions. Of the 31 ESX-1 GOI proteins, 11 contained at least one IDR, defined in this study by both a low predicted local distance difference test (pLDDT) confidence score and a high IDR prediction score (40) (Fig. S6–S9; Table S5). To filter out flexible loops connecting folded domains, stretches containing a minimum of 16 amino acids were kept. Out of those 11 IDR-containing proteins, 7 substrates (PPE68 and EspB) and peripheral ones (EspA, EspE, EspI, EspJ, and EspK) contain large IDR stretches (between ~70 and ~290 residues). By quantifying the relative nSNP content of those regions, we further split them into sub-regions of high and low polymorphism. As such, we identified a single conserved (~11% SNV) IDR for EspK, and polymorphic-only IDRs for EspA, EspJ, and PPE68; EspE, EspI, and EspB displayed both kinds (Fig. 5). IDRs harboring no nSNPs (in EspI and EspB) suggest a conserved role for these regions, as exemplified by the entirely nSNP-free IDR identified in EspB (residues 262–335) between the folded N-terminal domain (1–269) and the MycP1 cleavage site [Ala358/Ser359 (41)] that promotes the oligomerization of the protein (42, 43).

### Quaternary structures

To gain insight into the fidelity of interfaces for protein complexes utilized by the T7SS in pathogenic mycobacteria, we conducted a thorough examination of nSNPs within the context of quaternary structures. We employed the PISA interface tool (44) to scrutinize known complex interfaces, using a BSA score > 0 as an indicator of predicted interaction sites. For every complex interface in *Mycobacterium tuberculosis* (Mtb), we quantified the number of nSNPs associated with each interacting residue.

To begin, we identified nSNPs within experimentally verified regions where multiple ESX-1 substrates have been documented to interact (29, 42, 45–47). Focusing on the primary substrate complex transported by the ESX-1 system, we emphasize the conservation within the interaction sites between EsxA and EsxB. Within these sites, 19 amino acids participate in their interaction. Remarkably, only two interacting residues from each protein exhibited counts of two to three detected nSNPs at each position. To put this into perspective, these SNP occurrences were observed in just nine isolates out of a pool exceeding 32,000 (as detailed in Table S7), with a notable hotspot nSNP located at EsxB (E68K, 2,478 nSNPs).

When we focused our analysis on the self-interactions of EspB and PhoR oligomers, we discovered fewer than 50 nSNPs in less than 25% of the interacting residues (as detailed in Tables S8 and S9). Moreover, within the 15 residues involved in the biologically validated interaction between EspK and EspB, only five residues exhibited nSNPs (with counts ranging from 1 to 15), while the remaining 10 residues showed no nSNPs across the pool of analyzed genomes. This high level of conservation in the residues mediating this interaction emphasizes the stability and fidelity of the EspK–EspB complex (as shown in Table S10).

**Fig 6:**
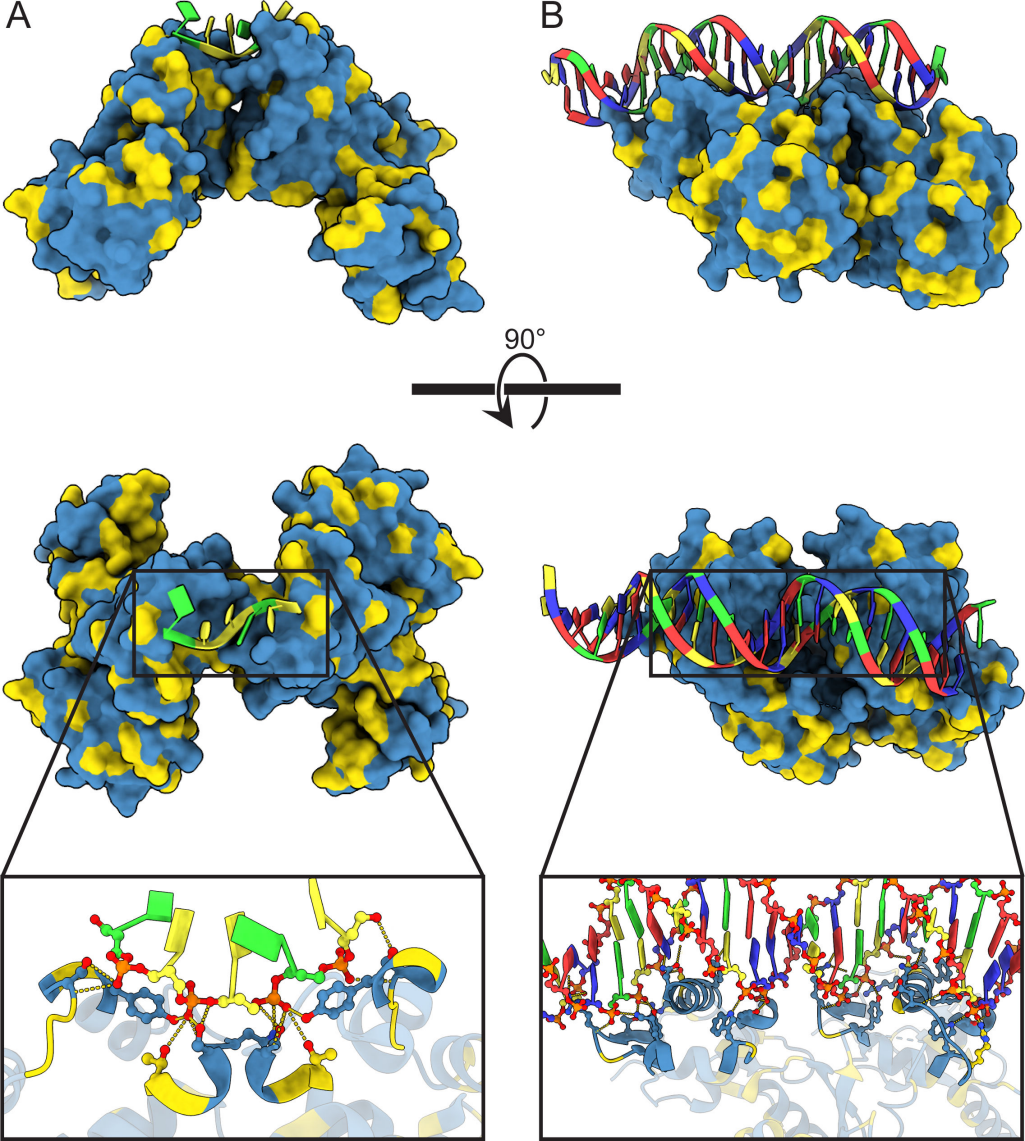
High level of conservation in the interfaces of EspR (A) and PhoP (B) that interact with DNA. nSNPs were mapped and colour-coded onto the experimentally derived structural models for the ESX-1 transcription regulators EspR (PDB: 3QYX) and PhoP (PDB: 5ED4). Colour code: blue = 0 nSNP, yellow ≥ 1 nSNP in 32,000 clinical isolates.

Next, we turned our attention to regions of DNA–protein interaction, specifically focusing on regulators EspR and PhoP (as depicted in Fig. 6). Remarkably, we identified complete conservation in all 11 interaction sites, comprising seven residues in the PhoP–DNA interaction and four residues in the EspR–DNA interaction (as detailed in Tables S11 and S12). This conservation strongly signifies the functional importance of these interaction sites for precise gene regulation within the context of Mtb’s virulence mechanisms.

Finally, we analyzed the conservation of the cysteines, present in 22 of the 31 ESX-1 proteins (Table S13): these rare, highly reactive residues play crucial roles in modulating intra- and intermolecular protein stability. We examined the cysteines involved in the formation of disulfide bonds in the periplasmic domain of the core components EccB1 (Cys150-Cys345), MycP1 (Cys49-Cys118 and Cys204-Cys242), and the secreted peripheral protein EspE (Cys114-Cys170). A single nSNP was identified within the disulfide bridge of EccB1. We also inspected the cysteines involved in the probable homo- or hetero-multimerization of the secreted peripheral components EspA, C, and D (Cys138, Cys48, and Cys174, respectively). EspA only harbored nSNPs at that position in the form of a mutation hotspot (640 nSNPs). Mutation of this position has been linked to the lack of dimerization of EspA and a general decrease in virulence but is not associated with a loss of ESX-1-mediated secretion (48), highlighting the probable role of EspA in the correct delivery of the virulence factors to the host. Regarding cytoplasmic components, the reducing conditions imposed intracellularly do not favor the formation of such disulfide bridges. In general, the cysteines belonging to the cytosolic components are generally well conserved (11 or less nSNPs), except for Cys8 of EspM (25 nSNPs), Cys729 of EspK (20,242 nSNPs), and Cys53 of WhiB6 (76 nSNPs). The latter may form a [4Fe-4S] cluster (Pfam: PF02467)—together with Cys34, Cys56, and Cys62—seemingly involved in sensing the redox conditions within the mycobacterial cytosol and essential in downregulating the secretion of EsxA and EsxB during the innate immune response to mycobacterial infection (49). The general lack of nSNPs detected at the cysteine positions underscores the critical significance of their conservation for the proper activity of ESX-1 at both structural and functional levels.

## DISCUSSION

The Mtbc genome is very conserved between lineages and species. Over a ~4.4 Mb genome, we identified a grand total of ~800,000 SNPs (positions, ~18% of the genome) representing nSNPs over the 32,399 isolates, of which half were singletons (a single nSNP identified per SNP position). In comparison, the ESX-1 GOI displays an average of 13% ± 3.7% SNPs (range 4%–21%), indicating those 31 genes undergo different selection pressures. Within the 31 GOI, the five most mutated genes encoding EccB1, EccD1, EspJ, PhoR, and WhiB6 (all with >50% of mutated amino-acid positions) may be under a stronger positive selection pressure than the other 28 genes. Co-evolution analyses may reveal new interaction partners for these proteins. Despite clonal expansion being the major force behind the spreading of new Mtb variants (50), the maximum nSNP counts for ESX-1 proteins EspC and EsxA are still fairly low (13 and 17, respectively), and the SNPs identified in these genes are mostly singletons; this might be related to their essential role in Mtb’s parasitic lifestyle, with their respective genes undergoing a strong negative selection.

The core complex proteins exhibited the highest degree of variation as a group within the ESX-1 proteins. As these mutations were not detrimental to the virulence of Mtbc *in vivo*, we can conclude they did not alter the structural integrity of the machinery nor the interactions between the different components sustaining its function. While the structural integrity of the machinery may not be easily compromised by single point mutations, the interactions between the different partners of this intricate complex might be disrupted. However, it must be noted that the disulfide-bridge-forming cysteines showed a very high degree of conservation (i.e., a single nSNP identified over 10 positions) that underscores the importance of maintaining structural integrity for the components involved. The other cysteines in ESX-1 were more prone to mutations (55% SNPs over 47 positions) as exemplified by the ones found in the global regulator WhiB6. We identified mutants at each position, four of which were involved in the formation of a putative iron-sulfur cluster 4Fe:4S. This redox-reactive cluster is heavily involved in the regulation of ESX-1-mediated virulence. *M. marinum* mutant exhibited reduced ESX-1-mediated, contact-dependent hemolysis *in vitro*, concomitant to reduced secretion levels of EsxA and EsxB (49). A detailed analysis of the 89 mutants identified in this study may help unveil new regulation networks involved in maintaining virulence in those isolates. As well, co-evolution network analyses may help in identifying mutation pairs responsible for intra- and inter-molecular epistasis involved in stabilizing the various variants at other positions. An experimentally determined structure of the ESX-1 inner membrane complex would also provide a suitable framework for a detailed analysis of these mutations.

Our analyses revealed ample sequence conservation in the known interaction and active sites of ESX-1 proteins, such as the ATPase domains of the core complex. Most proteins have signature sequences or motifs that are characteristic of protein families and represent an important feature in the protein structure or function. The Walker B motifs were indeed conserved in the ESX-1 ATPases. It is noteworthy that even the ATPase 2 and 3 domains of EccCb1 are relatively conserved even though these domains may not be catalytically active (51), as suggested by the orientation of the involved residues in the experimental *Thermomonospora curvata* EccC structural model, and the discovery of ATP molecules bound to these domains (51). However, those observations may not be relevant to EccCb proteins of the Mtbc strains as we have evidence of ATPase activity in EccCb1 from *M. tuberculosis* H37Rv (B.C. de Jong, unpublished data). Moreover, several putative sites for post-translational glycosylation and phosphorylation identified in the ESX-1-associated proteins displayed considerate conservation, thus highlighting their relevance as diagnostics or drug and vaccine targets.

Additionally, some IDRs also proved to be highly conserved, harboring only a few nSNPs. While several IDP-specialized prediction tools have been developed in the past two decades, AlphaFold has become a surprisingly efficient tool at identifying IDRs (long amino-acid stretches exhibiting low pLDDT confidence score) (52). We located a large proportion of such putative IDRs in 11 ESX-1 proteins, in which four substrates/peripheral proteins displayed highly conserved, long amino-acid IDR stretches. The functions of IDRs are not tied to their proper spatial folding but rather to their lack thereof. These natively unfolded proteins may undergo partial conformational arrangement upon binding of specific ligands (nucleic acids, co-factors, membranes, and proteins) and often play a scaffolding role in the establishment of protein complexes. This high conservation level implies an essential nature of these regions for the intracellular lifestyle of parasitic Mtb and—together with their secreted nature—their possible involvement with host factors. In addition, a strong immuno-modulator role can specifically be attributed to the IDRs encoded by the proteins of the PE/PPE/PE_PGRS families. Those low-complexity regions have been shown to be prone to accumulate polymorphisms and may act as decoy antigens, thus helping Mtb to evade immune surveillance by the host (53). The different levels of conservation observed for the different IDRs identified within the peripheral and secreted substrates of the ESX-1 machinery imply those regions may fulfill different roles, with the polymorphic IDRs acting on immune evasion, while the highly conserved ones assume functions essential to the survival and pathogenesis of Mtb, probably involving interactions with some host factors that may not allow for molecular epistasis between the host and its parasite. This is perfectly exemplified by EspB’s first IDR region (amino acids ~260–340) that is thought to interact with phosphatidic acid (PA) and, to a lesser extent, with phosphatidylserine (PS) (54). Those phospholipids are abundant in the phagosome membrane and are involved in its maturation into bacteria-killing phagolysosomes. A major role of EspB in Mtb virulence resides in delaying the phagosome maturation, and it has been proposed that the unstructured residues following the folded N-terminal domain partially fold to bind on the surface of biological membranes containing negatively charged phospholipids such as PA and PS, thus interfering with their trafficking (42, 55). The lack of nSNPs within that first EspB IDR supports this hypothesis, showing its essential role in Mtb virulence. The identification and characterization of other putative IDR-interacting host factors may require the use of tailored experimental strategies, such as microscopic co-localization of the interacting IDRs with the host ultrastructures, the direct pulldown of those factors from host cellular extracts using the IDRs of interest as baits, or the use of microarray screens. Unexpectedly, when analyzing the amino acid sequence of EspB (42), EspK (56), and EspI IDRs (data not shown) in other species, we noticed that these are the least conserved regions in the protein, only the nature of the amino acids is conserved, i.e., high content level of prolines and other nonpolar residues. The meaning of this observation will have to be further investigated, but it is possible that it is only important to maintain the physical chemistry properties of these IDRs between species, while in Mtb there are signatures with specific functions that cannot be modified.

In contrast to conserved regions, mutation hotspots seem to occur mostly at places on the protein surface for which no interactions have been reported so far. The observed clustering of nSNPs to a single amino acid was not surprising (Fig. 2), as surface areas (where no interactions occur) are known to be prone to polymorphisms. The hotspots suggest that these regions can vary without functional consequence and thus have no evolutionary pressure to remain stable.

Overall, our analysis of nSNP counts in specific interactions and post-translational modification sites within the ESX-1 genes of Mtb provides valuable insights into the conservation and genetic diversity within these critical protein–protein and protein–DNA interfaces. These findings contribute to our understanding of the functional significance of these interactions and their potential implications for Mtb’s pathogenicity. Moreover, we showed here the potential of this approach to identify clinically relevant molecular targets in the presence of both highly conserved IDRs and motifs essential to the virulence, which should be further taken up by the Mtb community as potential targets for new vaccines. In this study, the use of a limited number of sequenced genomes from clinical isolates, relative to the size of the Mtb genome, did not allow for a true identification of essential protein regions at amino-acid resolution; however, it can be expected that the wider adoption of WGS as a diagnostics tool will soon allow the gathering of sufficient data to reach this goal and better understand the dynamics of Mtb virulence outside the laboratory.

Conserved areas in a pathogen genome are good targets for a vaccine, as vaccine efficacy requires both adequate immune coverage (adaptive immune mechanisms in people of diverse genetic backgrounds must be able to generate immunity against the vaccine) and vaccine epitopes against which immunity is induced must be conserved among variants of the pathogen of interest. Nemes et al. (57) compared nSNPs’ distribution in the Mtb genome divided into three gene sets, including “essential genes,” “non-essential genes,” and “antigens.” Essential genes were defined as transposon insertion mutants that were defective for the ability to grow on a solid medium. Antigens were defined based on the presence of experimentally confirmed human T-cell epitopes. The observation that the known human T-cell epitopes are hyperconserved suggests that the bacteria actually benefit from T-cell recognition. Given the lengthy co-evolution of MTB and humans, it is yet unclear if the identification of hyperconserved epitopes yields ideal vaccine targets or favors MTBs’ immune recognition leading to increased cavitation and ongoing transmission.

## MATERIALS AND METHODS

### Whole-genome sequencing and phylogenetic analysis of 967 clinical isolates

A total of 967 genomes of clinical isolates from the Mtbc (*n* = 961; L1–L8) and from *M. bovis* (*n* = 6) were included, from Bangladesh and Gambia (58–60) (Supplemantary Table). We used this data set as the initial backbone phylogeny for the rest of the analysis. The semi-automated Mtbseq pipeline was used for read mapping and variant detection (61). The output of Mtbseq was used to generate an SNP alignment using an in-house Python script (https://github.com/alxndravc/ESX-1-MS). Based on this SNP alignment, a maximum likelihood tree was built using RaxML-NG (62) with a GTR + CAT model of evolution and 100 bootstraps; *M. canettii* was added as an outgroup. The different phylogenetic lineages were visualized using the online interactive Tree Of Life tool (63).

### Phylogenetic analysis of 31,428 publicly available Mtbc isolates

We used an SNP barcode (64) to type a collection of 31,428 WGS Mtbc isolates downloaded from NCBI into Mtbc lineage and sub-lineage. We excluded isolates that were missing 10% of SNP sites, were not typed as belonging to Mtbc L1–6, or were typed as L4 but were not typed further with an L4 sub-lineage. We split the 31,428 isolates into eight groups based on genetic similarity, five groups corresponding to global L1, L2, L3, L5, and L6, and three groups for lineage 4 (i.e., L4.1.12). To generate phylogenies for each of these groups, we first merged VCF files of the isolates in each group with bcftools (65). We then removed repetitive, antibiotic resistance, and low-coverage regions (64). We generated a multi-sequence FASTA alignment from the merged VCF file with vcf2phylip (version 1.5). Finally, we constructed the phylogenetic trees for each group with IQ-TREE 1.6.12 (66). We used the *mset* option to restrict model selection to GTR + CAT models and selected the GTR + F + I + R model for the six isolate groups corresponding to L1–L4 and implemented the automatic model selection with ModelFinder Plus (67) for the isolate groups corresponding to L5 and L6.

### SNP catalog

An in-house Python script was used to count the unique SNPs present within each isolate group (stratified by global lineage) within the ESX-1 GOI (https://github.com/alxndravc/ESX-1-MS). To calculate SNP frequency per 1 kb, the number of unique SNP locations per gene was multiplied by 1,000 bp and divided by their respective gene length. The same methodology was used on the 31,000 WGS isolates from NCBI. The genes were divided into four groups: machinery, substrates, regulatory, and peripheral (i.e., genes not belonging to any of the other three categories) (Table S1).

### SNPPar analysis homoplasy

The resulting phylogenetic tree from the 967 clinical isolates was used in SNPPar along with the SNP data set to obtain the mutation events across all Mtbc lineages. To screen for convergent SNP sites in the alignment, SNPPar was used. Based on the provided phylogenetic tree, SNPPar searches for SNPs that are the same mutation (e.g*.,* C, G) at the same position in two or more unrelated isolates or different mutations that result in the same base (e.g*.,* C, G, A, G) in the same position. It also detects revertant mutations back to the ancestral state (e.g., C G C) (68). We used the default settings of SNPPar, which is a TreeTime for ancestral state reconstruction. As input, the phylogenies mentioned above were used together with the H37Rv reference genome in GenBank format (NC_000962.3) and an SNP position file. On the large data set of 31,428 publicly available samples, SNPPar was run eight times on eight independent sets of isolates corresponding to eight genetic backgrounds (L1–L6). Lineage sample counts are as follows: L1: 2,815; L2: 8,090; L3: 3,398; L4A: 5,839; L4B: 6,958; L4C: 4,134; L5: 98; and L6: 96.

### Mapping of the nSNPs on the ESX-1 predicted models

AlphaFold2-generated protein 3D models were collected from the EBI/AlphaFold collection of models built upon the UniProt database (version 4, last accessed on 20 July 2022, available at https://alphafold.ebi.ac.uk), using their corresponding UniProt reference. For each model, pLDDT scores per amino-acid position were extracted from the coordinates files (b-factor column), and nSNP counts per amino acid were assigned into corresponding attribute files prior to their rendering with UCSF Chimera 1.15 (69) and/or ChimeraX 1.6.1 (70) with the following coloring scheme: blue for 0 or yellow >0 SNPs, red for the 1% outliers (>300 SNPs).

### Modeling of the ESX-1 core complex

To this date, no experimental structural model of the ESX-1 core complex (EccB1/Ca1/Cb1/D1/E1) has been deciphered; however, the structures of the homologous complexes ESX-3 of *Mycobacterium smegmatis* (17) and ESX-5 of *M. tuberculosis* (18) were recently solved by cryo-SPA (single particle averaging) electron microscopy. The overall high homology levels observed between the components of ESX-1 and those of ESX-3 and ESX-5 allowed us, with the help of AlphaFold2-generated models, to propose a composite predicted model of the whole ESX-1 inner-membrane core machinery. The general procedure consisted first of retrieving (i) the AF2-generated models of the ESX-1 core components through their respective Uniprot references and (ii) the atomic models of the ESX-3 and ESX-5 core complex components determined from the experimental cryo-SPA experiments (PDB: 6SGX, 6SGZ, and EMD-10187, and PDB: 7NPR and EMD-12517, respectively, obtained from https://www.rcsb.org and https://www.ebi.ac.uk/emdb; see Table S14). Then, using UCSF Chimera 1.15, domain orientation in the core ESX-1 AF2-generated models was corrected by manually isolating the individual domains, structurally aligning them to their ESX-3/5 counterparts, and reassembling them with the program Modeller 9.20 (71) (available at https://salilab.org/modeller). Finally, the tweaked ESX-1 models were assembled into a composite model using the whole core ESX-3 and ESX-5 complexes as references: a protomer dimer containing two copies of EccB1, EccCa1, EccCb1, and EccE1, four copies of EccD1, and one copy of MycP1 linking the protomers was first assembled; the larger complex was deduced by trimerization of this protomer dimer (C3 symmetry).

Specific modifications were applied to the ESX-1 initial models as follows: for EccB1, two different models were derived from the initial AF2 model to account for the difference in the orientation of the N-terminal helices (located in the cytoplasm and the inner membrane, respectively) found in the protomers of ESX-3 and ESX-5. Regarding EccCa1 and EccCb1, the ESX-3 and ESX-5 cryo-SPA density maps do not offer a sufficient resolution for the modeling of the EccC3 and EccC5 cytoplasmic regions; the relative orientation of the EccCb1 sub-unit toward EccCa1 was deduced by a structural alignment using the experimentally deduced model of the EccC cytoplasmic domain from *T. curvata* (PDB: 4NH0), in which the different ATPase domains are physically linked through covalent bonding (51); remarkably, this solution allowed for the EccCb1 monomers to not overlap with each other but assemble in a ring-like structure of the N-terminal ATPase domains of EcCb1 in the final ESX-1 composite model. For the EccD1 dimer, the assembly of the C-terminal transmembrane domains used EccD3 (from PDB: 6SGZ) and EccD5 (from PDB: 7NPR) dimers as references; regarding the assembly of the N-terminal ubiquitin-like domain, the cyclin C2 dimer assembly identified by X-ray diffraction (72) (9PDB: 4KV2) was disregarded, instead the dimerization of that domain was modeled with EccD3 and EccD5 as references as well. Finally, the N-terminal hydrophobic alpha-helix of MycP1 was truncated to mimic the maturation of the signal peptide during trafficking, prior to adjusting the orientation of the C-terminal linker and membrane alpha-helix anchor using MycP5 as a reference (from PDB: 7NPR). The PhoR and MprB dimers were predicted using AlphaFold2-multimer (73).

### nSNPs vs pLDDT vs IDR plots

Putative IDRs of the 31 ESX-1 proteins of interest (POIs) were predicted in batch using the OdiNPred server (Prediction of Order and Disorder by evaluation of NMR data, https://st-protein.chem.au.dk/odinpred) without evolution; the disorder probability (DP) scores were retained for further processing. nSNP counts, pLDDT scores, and DP scores were assigned to each amino acid position, per POI, and DP scores were normalized to percentage values for plotting purposes. Figures were generated with SigmaPlot 12.5 (Systat Software): nSNPs’ counts per amino acid were displayed on a logarithmic scale (0.1–40,000, left axis), while pLDDT and DP scores per amino acid were displayed on a percentage scale (0–100, right axis).

### Expression and purification of EsxA-EsxB wild type and E68K mutant

Plasmid pET29-EsxB-EsxA-6xHis used for expressing wild-type EsxAB was a gift from Lalita Ramakrishnan. The single point mutation of E68K was generated using site-directed mutagenesis strategy and confirmed by sequencing. The conditions for the expression of both wild type and mutant were used as described by Conrad et al. (74). Proteins were purified using Ni^2+^-Nitrilotriacetic acid affinity resin (Qiagen) and a size exclusion chromatography Superdex 75 3.2/300 GL column (GE Healthcare Life Science) in 20 mM Tris-HCl (pH 8) and 150 mM NaCl. Purity assessment was done by SDS-PAGE and protein identification by mass spectrometry.

### Quaternary structure analysis

To count SNPs in active sites residues, we used the PISA interface tool (https://www.ebi.ac.uk/pdbe/pisa/) to search for residues with buried surface value (BSA) > 0. Furthermore, the putative effects of point mutations on protein stability were assessed with FoldX (75) (https://foldxsuite.crg.eu/).

### Sanger sequencing of *espI*

To validate the two *espI* silent SNPs (Pro134/135Pro), primers were designed for Sanger sequencing on the DNA extracts of available mutant isolates (i.e., containing the SNPs) and phylogenetically closely related wild-type isolates. A list of used genetic isolates and primer sequences can be found in Table S2.

### Data visualization

Circos and histogram plots were generated using R statistical software, version 4.0.2 (76). IDR plots were drawn with SigmaPlot. Protein structures were generated using UCSF ChimeraX, version 1.6.1 (70).

## Data Availability

*M. tuberculosis* whole genome sequencing data were collected from NCBI and are publicly available (see Materials and Methods).

## SUPPLEMENTAL MATERIAL

Fig. S1 spectrum.03816-23-s0001.tiff Supplemental Figure 1.

Fig. S2 spectrum.03816-23-s0002.tif Supplemental Figure 2.

Fig. S3 spectrum.03816-23-s0003.tiff Supplemental Figure 3.

Fig. S4 spectrum.03816-23-s0004.tiff Supplemental Figure 4.

Fig. S5 spectrum.03816-23-s0005.tiff Supplemental Figure 5.

Fig. S6 spectrum.03816-23-s0006.tiff Supplemental Figure 6.

Fig. S7 spectrum.03816-23-s0007.tif Supplemental Figure 7.

Fig. S8 spectrum.03816-23-s0008.tif Supplemental Figure 8.

Fig. S9 spectrum.03816-23-s0009.tiff Supplemental Figure 9.

Supplementary Material spectrum.03816-23-s0010.xlsx Tables S1 to S15.

ASM does not own the copyrights to Supplemental Material that may be linked to, or accessed through, an article. The authors have granted ASM a non-exclusive, world-wide license to publish the Supplemental Material files. Please contact the corresponding author directly for reuse.

## References

[1] Böddinghaus B, Rogall T, Flohr T, Blöcker H, Böttger EC. 1990. Detection and identification of mycobacteria by amplification of rRNA. J Clin Microbiol 28:1751–1759. doi:10.1128/jcm.28.8.1751-1759.1990 2203812 PMC268042

[2] Sreevatsan S, Pan X, Stockbauer KE, Connell ND, Kreiswirth BN, Whittam TS, Musser JM. 1997. Restricted structural gene polymorphism in the Mycobacterium tuberculosis complex indicates evolutionarily recent global dissemination. Proc Natl Acad Sci U S A 94:9869–9874. doi:10.1073/pnas.94.18.9869 9275218 PMC23284

[3] Achtman M, Wagner M. 2008. Microbial diversity and the genetic nature of microbial species. Nat Rev Microbiol 6:431–440. doi:10.1038/nrmicro1872 18461076

[4] Wiens KE, Woyczynski LP, Ledesma JR, Ross JM, Zenteno-Cuevas R, Goodridge A, Ullah I, Mathema B, Djoba Siawaya JF, Biehl MH, Ray SE, Bhattacharjee NV, Henry NJ, Reiner RC Jr, Kyu HH, Murray CJL, Hay SI. 2018. Global variation in bacterial strains that cause tuberculosis disease: a systematic review and meta-analysis. BMC Med 16:196. doi:10.1186/s12916-018-1180-x 30373589 PMC6206891

[5] Andersen P, Andersen AB, Sørensen AL, Nagai S. 1995. Recall of long-lived immunity to Mycobacterium tuberculosis infection in mice. J Immunol 154:3359–3372. 7897219

[6] Brodin P, Rosenkrands I, Andersen P, Cole ST, Brosch R. 2004. ESAT-6 proteins: protective antigens and virulence factors? Trends Microbiol 12:500–508. doi:10.1016/j.tim.2004.09.007 15488391

[7] van der Wel N, Hava D, Houben D, Fluitsma D, van Zon M, Pierson J, Brenner M, Peters PJ. 2007. M. tuberculosis and M. leprae Translocate from the phagolysosome to the cytosol in myeloid cells. Cell 129:1287–1298. doi:10.1016/j.cell.2007.05.059 17604718

[8] Xu J, Laine O, Masciocchi M, Manoranjan J, Smith J, Du SJ, Edwards N, Zhu X, Fenselau C, Gao LY. 2007. A unique Mycobacterium ESX-1 protein co-secretes with CFP-10/ESAT-6 and is necessary for inhibiting phagosome maturation. Mol Microbiol 66:787–800. doi:10.1111/j.1365-2958.2007.05959.x 17908204

[9] Houben D, Demangel C, van Ingen J, Perez J, Baldeón L, Abdallah AM, Caleechurn L, Bottai D, van Zon M, de Punder K, van der Laan T, Kant A, Bossers-de Vries R, Willemsen P, Bitter W, van Soolingen D, Brosch R, van der Wel N, Peters PJ. 2012. ESX-1-mediated translocation to the cytosol controls virulence of mycobacteria. Cell Microbiol 14:1287–1298. doi:10.1111/j.1462-5822.2012.01799.x 22524898

[10] Tiwari S, Casey R, Goulding CW, Hingley-Wilson S, Jacobs WR. 2019. Infect and inject: how Mycobacterium tuberculosis exploits its major virulence-associated type VII secretion system, ESX-1. Microbiol Spectr 7. doi:10.1128/microbiolspec.BAI-0024-2019 PMC6698389 31172908

[11] Mahairas GG, Sabo PJ, Hickey MJ, Singh DC, Stover CK. 1996. Molecular analysis of genetic differences between Mycobacterium bovis BCG and virulent M. bovis. J Bacteriol 178:1274–1282. doi:10.1128/jb.178.5.1274-1282.1996 8631702 PMC177799

[12] Lewis KN, Liao R, Guinn KM, Hickey MJ, Smith S, Behr MA, Sherman DR. 2003. Deletion of Rd1 from Mycobacterium tuberculosis mimics bacille Calmette-Guerin attenuation. J Infect Dis 187:117–123. doi:10.1086/345862 12508154 PMC1458498

[13] Orgeur M, Frigui W, Pawlik A, Clark S, Williams A, Ates LS, Ma L, Bouchier C, Parkhill J, Brodin P, Brosch R. 2021. Pathogenomic analyses of Mycobacterium microti, an ESX-1-deleted member of the Mycobacterium tuberculosis complex causing disease in various hosts. Microb Genom 7:000505. doi:10.1099/mgen.0.000505 33529148 PMC8208694

[14] Tekaia F, Gordon SV, Garnier T, Brosch R, Barrell BG, Cole ST. 1999. Analysis of the proteome of Mycobacterium tuberculosis in silico. Tuber Lung Dis 79:329–342. doi:10.1054/tuld.1999.0220 10694977

[15] Jagielski T, Borówka P, Bakuła Z, Lach J, Marciniak B, Brzostek A, Dziadek J, Dziurzyński M, Pennings L, van Ingen J, Žolnir-Dovč M, Strapagiel D. 2019. Genomic insights into the Mycobacterium kansasii complex: an update. Front Microbiol 10:2918. doi:10.3389/fmicb.2019.02918 32010067 PMC6974680

[16] Gey van Pittius NC, Gamieldien J, Hide W, Brown GD, Siezen RJ, Beyers AD. 2001. The ESAT-6 gene cluster of Mycobacterium tuberculosis and other high G+C gram-positive bacteria. Genome Biol 2. doi:10.1186/gb-2001-2-10-research0044 PMC57799 11597336

[17] Famelis N, Rivera-Calzada A, Degliesposti G, Wingender M, Mietrach N, Skehel JM, Fernandez-Leiro R, Böttcher B, Schlosser A, Llorca O, Geibel S. 2019. Architecture of the mycobacterial type VII secretion system. Nature 576:321–325. doi:10.1038/s41586-019-1633-1 31597161 PMC6914368

[18] Bunduc CM, Fahrenkamp D, Wald J, Ummels R, Bitter W, Houben ENG, Marlovits TC. 2021. Structure and dynamics of a mycobacterial type VII secretion system. Nature 593:445–448. doi:10.1038/s41586-021-03517-z 33981042 PMC8131196

[19] Baek M, DiMaio F, Anishchenko I, Dauparas J, Ovchinnikov S, Lee GR, Wang J, Cong Q, Kinch LN, Schaeffer RD, et al.. 2021. Accurate prediction of protein structures and interactions using a three-track neural network. Science 373:871–876. doi:10.1126/science.abj8754 34282049 PMC7612213

[20] Jumper J, Evans R, Pritzel A, Green T, Figurnov M, Ronneberger O, Tunyasuvunakool K, Bates R, Žídek A, Potapenko A, et al.. 2021. Highly accurate protein structure prediction with AlphaFold. Nature 596:583–589. doi:10.1038/s41586-021-03819-2 34265844 PMC8371605

[21] Brosch R, Gordon SV, Garnier T, Eiglmeier K, Frigui W, Valenti P, Dos Santos S, Duthoy S, Lacroix C, Garcia-Pelayo C, Inwald JK, Golby P, Garcia JN, Hewinson RG, Behr MA, Quail MA, Churcher C, Barrell BG, Parkhill J, Cole ST. 2007. Genome plasticity of BCG and impact on vaccine efficacy. Proc Natl Acad Sci U S A 104:5596–5601. doi:10.1073/pnas.0700869104 17372194 PMC1838518

[22] Brosch R, Gordon SV, Marmiesse M, Brodin P, Buchrieser C, Eiglmeier K, Garnier T, Gutierrez C, Hewinson G, Kremer K, Parsons LM, Pym AS, Samper S, van Soolingen D, Cole ST. 2002. A new evolutionary scenario for the Mycobacterium tuberculosis complex. Proc Natl Acad Sci U S A 99:3684–3689. doi:10.1073/pnas.052548299 11891304 PMC122584

[23] Peters JS, Ismail N, Dippenaar A, Ma S, Sherman DR, Warren RM, Kana BD. 2020. Genetic diversity in Mycobacterium tuberculosis clinical isolates and resulting outcomes of tuberculosis infection and disease. Annu Rev Genet 54:511–537. doi:10.1146/annurev-genet-022820-085940 32926793 PMC12646202

[24] Wirth T, Hildebrand F, Allix-Béguec C, Wölbeling F, Kubica T, Kremer K, van Soolingen D, Rüsch-Gerdes S, Locht C, Brisse S, Meyer A, Supply P, Niemann S. 2008. Origin, spread and demography of the Mycobacterium tuberculosis complex. PLoS Pathog 4:e1000160. doi:10.1371/journal.ppat.1000160 18802459 PMC2528947

[25] Vargas R, Luna MJ, Freschi L, Marin M, Froom R, Murphy KC, Campbell EA, Ioerger TR, Sassetti CM, Farhat MR. 2023. Phase variation as a major mechanism of adaptation in Mycobacterium tuberculosis complex. Proc Natl Acad Sci U S A 120:e2301394120. doi:10.1073/pnas.2301394120 37399390 PMC10334774

[26] Walker JE, Saraste M, Runswick MJ, Gay NJ. 1982. Distantly related sequences in the alpha- and beta-subunits of ATP synthase, myosin, kinases and other ATP-requiring enzymes and a common nucleotide binding fold. EMBO J 1:945–951. doi:10.1002/j.1460-2075.1982.tb01276.x 6329717 PMC553140

[27] Chiner-Oms Á, Berney M, Boinett C, González-Candelas F, Young DB, Gagneux S, Jacobs WR, Parkhill J, Cortes T, Comas I. 2019. Genome-wide mutational biases fuel transcriptional diversity in the Mycobacterium tuberculosis complex. Nat Commun 10:3994. doi:10.1038/s41467-019-11948-6 31488832 PMC6728331

[28] Dogan S, Cilic A, Marjanovic D, Kurtovic-Kozaric A. 2017. Detection of cytosine and CpG density in proto-oncogenes and tumor suppressor genes in promoter sequences of acute myeloid leukemia. Nucleosides, Nucleotides & Nucleic Acids 36:302–316. doi:10.1080/15257770.2017.1279738 28323522

[29] Poulsen C, Panjikar S, Holton SJ, Wilmanns M, Song YH. 2014. WXG100 protein superfamily consists of three subfamilies and exhibits an alpha-helical C-terminal conserved residue pattern. PLoS One 9:e89313. doi:10.1371/journal.pone.0089313 24586681 PMC3935865

[30] Renshaw PS, Lightbody KL, Veverka V, Muskett FW, Kelly G, Frenkiel TA, Gordon SV, Hewinson RG, Burke B, Norman J, Williamson RA, Carr MD. 2005. Structure and function of the complex formed by the tuberculosis virulence factors CFP-10 and ESAT-6. EMBO J 24:2491–2498. doi:10.1038/sj.emboj.7600732 15973432 PMC1176459

[31] Allemand JF, Maier B, Smith DE. 2012. Molecular motors for DNA translocation in prokaryotes. Curr Opin Biotechnol 23:503–509. doi:10.1016/j.copbio.2011.12.023 22226958 PMC3381886

[32] Zhang M, Chen JM, Sala C, Rybniker J, Dhar N, Cole ST. 2014. EspI regulates the ESX-1 secretion system in response to ATP levels in Mycobacterium tuberculosis. Mol Microbiol 93:1057–1065. doi:10.1111/mmi.12718 25039394 PMC4150839

[33] Chen H, Wang H, Sun T, Tian S, Lin D, Guo C. 2016. Recombinant preparation and functional studies of EspI ATP binding domain from Mycobacterium tuberculosis. Protein Expr Purif 123:51–59. doi:10.1016/j.pep.2016.03.009 27017992

[34] Birhanu AG, Yimer SA, Kalayou S, Riaz T, Zegeye ED, Holm-Hansen C, Norheim G, Aseffa A, Abebe M, Tønjum T. 2019. Ample glycosylation in membrane and cell envelope proteins may explain the phenotypic diversity and virulence in the Mycobacterium tuberculosis complex. Sci Rep 9:2927. doi:10.1038/s41598-019-39654-9 30814666 PMC6393673

[35] Singh PK, Saxena R, Tiwari S, Singh DK, Singh SK, Kumari R, Srivastava KK. 2015. RD-1 encoded EspJ protein gets phosphorylated prior to affect the growth and intracellular survival of mycobacteria. Sci Rep 5:12717. doi:10.1038/srep12717 26228622 PMC4521147

[36] Xing D, Ryndak MB, Wang L, Kolesnikova I, Smith I, Wang S. 2017. Asymmetric structure of the dimerization domain of PhoR, a sensor kinase important for the virulence of Mycobacterium tuberculosis. ACS Omega 2:3509–3517. doi:10.1021/acsomega.7b00612 28782049 PMC5537716

[37] Zahrt TC, Wozniak C, Jones D, Trevett A. 2003. Functional analysis of the Mycobacterium tuberculosis MprAB two-component signal transduction system. Infect Immun 71:6962–6970. doi:10.1128/IAI.71.12.6962-6970.2003 14638785 PMC308901

[38] Aderinwale T, Bharadwaj V, Christoffer C, Terashi G, Zhang Z, Jahandideh R, Kagaya Y, Kihara D. 2022. Real-time structure search and structure classification for AlphaFold protein models. Commun Biol 5:316. doi:10.1038/s42003-022-03261-8 35383281 PMC8983703

[39] Piovesan D, Monzon AM, Tosatto SCE. 2022. Intrinsic protein disorder and conditional folding in AlphaFoldDB. Protein Sci 31:e4466. doi:10.1002/pro.4466 36210722 PMC9601767

[40] Dass R, Mulder FAA, Nielsen JT. 2020. ODiNPred: comprehensive prediction of protein order and disorder. Sci Rep 10:14780. doi:10.1038/s41598-020-71716-1 32901090 PMC7479119

[41] Solomonson M, Huesgen PF, Wasney GA, Watanabe N, Gruninger RJ, Prehna G, Overall CM, Strynadka NCJ. 2013. Structure of the mycosin-1 protease from the mycobacterial ESX-1 protein type VII secretion system. J Biol Chem 288:17782–17790. doi:10.1074/jbc.M113.462036 23620593 PMC3682577

[42] Gijsbers A, Vinciauskaite V, Siroy A, Gao Y, Tria G, Mathew A, Sánchez-Puig N, López-Iglesias C, Peters PJ, Ravelli RBG. 2021. Priming mycobacterial ESX-secreted protein B to form a channel-like structure. Curr Res Struct Biol 3:153–164. doi:10.1016/j.crstbi.2021.06.001 34337436 PMC8313811

[43] Solomonson M, Setiaputra D, Makepeace KAT, Lameignere E, Petrotchenko EV, Conrady DG, Bergeron JR, Vuckovic M, DiMaio F, Borchers CH, Yip CK, Strynadka NCJ. 2015. Structure of EspB from the ESX-1 type VII secretion system and insights into its export mechanism. Structure 23:571–583. doi:10.1016/j.str.2015.01.002 25684576

[44] Krissinel E, Henrick K. 2007. Inference of macromolecular assemblies from crystalline state. J Mol Biol 372:774–797. doi:10.1016/j.jmb.2007.05.022 17681537

[45] Blasco B, Stenta M, Alonso-Sarduy L, Dietler G, Peraro MD, Cole ST, Pojer F. 2011. Atypical DNA recognition mechanism used by the EspR virulence regulator of Mycobacterium tuberculosis. Mol Microbiol 82:251–264. doi:10.1111/j.1365-2958.2011.07813.x 21883526

[46] Gijsbers A, Eymery M, Gao Y, Menart I, Vinciauskaite V, Siliqi D, Peters PJ, McCarthy A, Ravelli RBG. 2023. The crystal structure of the EspB-EspK virulence factor-chaperone complex suggests an additional type VII secretion mechanism in Mycobacterium tuberculosis. J Biol Chem 299:102761. doi:10.1016/j.jbc.2022.102761 36463964 PMC9811218

[47] He X, Wang L, Wang S. 2016. Structural basis of DNA sequence recognition by the response regulator PhoP in Mycobacterium tuberculosis. Sci Rep 6:24442. doi:10.1038/srep24442 27079268 PMC4832192

[48] Garces A, Atmakuri K, Chase MR, Woodworth JS, Krastins B, Rothchild AC, Ramsdell TL, Lopez MF, Behar SM, Sarracino DA, Fortune SM. 2010. EspA acts as a critical mediator of ESX1-dependent virulence in Mycobacterium tuberculosis by affecting bacterial cell wall integrity. PLoS Pathog 6:e1000957. doi:10.1371/journal.ppat.1000957 20585630 PMC2891827

[49] Chen Z, Hu Y, Cumming BM, Lu P, Feng L, Deng J, Steyn AJC, Chen S. 2016. Mycobacterial WhiB6 differentially regulates ESX-1 and the dos regulon to modulate granuloma formation and virulence in zebrafish. Cell Rep 16:2512–2524. doi:10.1016/j.celrep.2016.07.080 27545883

[50] Eldholm V, Balloux F. 2016. Antimicrobial resistance in Mycobacterium tuberculosis: the odd one out. Trends Microbiol 24:637–648. doi:10.1016/j.tim.2016.03.007 27068531

[51] Rosenberg OS, Dovala D, Li X, Connolly L, Bendebury A, Finer-Moore J, Holton J, Cheng Y, Stroud RM, Cox JS. 2015. Substrates control multimerization and activation of the multi-domain ATPase motor of type VII secretion. Cell 161:501–512. doi:10.1016/j.cell.2015.03.040 25865481 PMC4409929

[52] Tunyasuvunakool K, Adler J, Wu Z, Green T, Zielinski M, Žídek A, Bridgland A, Cowie A, Meyer C, Laydon A, et al.. 2021. Highly accurate protein structure prediction for the human proteome. Nature 596:590–596. doi:10.1038/s41586-021-03828-1 34293799 PMC8387240

[53] Sharma T, Alam A, Ehtram A, Rani A, Grover S, Ehtesham NZ, Hasnain SE. 2022. The Mycobacterium tuberculosis PE_PGRS protein family acts as an immunological decoy to subvert host immune response. Int J Mol Sci 23:525. doi:10.3390/ijms23010525 35008950 PMC8745494

[54] Chen JM, Zhang M, Rybniker J, Boy-Röttger S, Dhar N, Pojer F, Cole ST. 2013. Mycobacterium tuberculosis EspB binds phospholipids and mediates EsxA-independent virulence. Mol Microbiol 89:1154–1166. doi:10.1111/mmi.12336 23869560

[55] Sengupta N, Padmanaban S, Dutta S. 2023. Cryo-EM reveals the membrane-binding phenomenon of EspB, a virulence factor of the mycobacterial type VII secretion system. J Biol Chem 299:104589. doi:10.1016/j.jbc.2023.104589 36889587 PMC10140165

[56] Gijsbers A, Sánchez-Puig N, Gao Y, Peters PJ, Ravelli RBG, Siliqi D. 2021. Structural analysis of the partially disordered protein EspK from Mycobacterium tuberculosis. Crystals 11:18. doi:10.3390/cryst11010018

[57] Nemes E, Fiore-Gartland A, Boggiano C, Coccia M, D’Souza P, Gilbert P, Ginsberg A, Hyrien O, Laddy D, Makar K, McElrath MJ, Ramachandra L, Schmidt AC, Shororbani S, Sunshine J, Tomaras G, Yu WH, Scriba TJ, Frahm N, Bcg Correlates Pis Study Team MCPST. 2022. The quest for vaccine-induced immune correlates of protection against tuberculosis. Vaccine Insights 1:165–181. doi:10.18609/vac/2022.027 37091190 PMC10117634

[58] Comas I, Coscolla M, Luo T, Borrell S, Holt KE, Kato-Maeda M, Parkhill J, Malla B, Berg S, Thwaites G, Yeboah-Manu D, Bothamley G, Mei J, Wei L, Bentley S, Harris SR, Niemann S, Diel R, Aseffa A, Gao Q, Young D, Gagneux S. 2013. Out-of-Africa migration and neolithic coexpansion of Mycobacterium tuberculosis with modern humans. Nat Genet 45:1176–1182. doi:10.1038/ng.2744 23995134 PMC3800747

[59] Lempens P, Decroo T, Aung KJM, Hossain MA, Rigouts L, Meehan CJ, Van Deun A, de Jong BC. 2020. Initial resistance to companion drugs should not be considered an exclusion criterion for the shorter multidrug-resistant tuberculosis treatment regimen. Int J Infect Dis 100:357–365. doi:10.1016/j.ijid.2020.08.042 32829049 PMC7670168

[60] Ngabonziza JCS, Loiseau C, Marceau M, Jouet A, Menardo F, Tzfadia O, Antoine R, Niyigena EB, Mulders W, Fissette K, Diels M, Gaudin C, Duthoy S, Ssengooba W, André E, Kaswa MK, Habimana YM, Brites D, Affolabi D, Mazarati JB, de Jong BC, Rigouts L, Gagneux S, Meehan CJ, Supply P. 2020. A sister lineage of the Mycobacterium tuberculosis complex discovered in the African Great Lakes region. Nat Commun 11:2917. doi:10.1038/s41467-020-16626-6 32518235 PMC7283319

[61] Kohl TA, Utpatel C, Schleusener V, De Filippo MR, Beckert P, Cirillo DM, Niemann S. 2018. MTBseq: a comprehensive pipeline for whole genome sequence analysis of Mycobacterium tuberculosis complex isolates. PeerJ 6:e5895. doi:10.7717/peerj.5895 30479891 PMC6238766

[62] Stamatakis A. 2014. RAxML version 8: a tool for phylogenetic analysis and post-analysis of large phylogenies. Bioinformatics 30:1312–1313. doi:10.1093/bioinformatics/btu033 24451623 PMC3998144

[63] Letunic I, Bork P. 2021. Interactive tree of life (iTOL) V5: an online tool for phylogenetic tree display and annotation. Nucleic Acids Res 49:W293–W296. doi:10.1093/nar/gkab301 33885785 PMC8265157

[64] Freschi L, Vargas R, Husain A, Kamal SMM, Skrahina A, Tahseen S, Ismail N, Barbova A, Niemann S, Cirillo DM, Dean AS, Zignol M, Farhat MR. 2021. Population structure, biogeography and transmissibility of Mycobacterium tuberculosis. Nat Commun 12:6099. doi:10.1038/s41467-021-26248-1 34671035 PMC8528816

[65] Li H, Handsaker B, Wysoker A, Fennell T, Ruan J, Homer N, Marth G, Abecasis G, Durbin R, Genome Project Data Processing S. 2009. The sequence alignment/map format and SAMtools. Bioinformatics 25:2078–2079. doi:10.1093/bioinformatics/btp352 19505943 PMC2723002

[66] Nguyen L-T, Schmidt HA, von Haeseler A, Minh BQ. 2015. IQ-TREE: a fast and effective stochastic algorithm for estimating maximum-likelihood phylogenies. Mol Biol Evol 32:268–274. doi:10.1093/molbev/msu300 25371430 PMC4271533

[67] Kalyaanamoorthy S, Minh BQ, Wong TKF, von Haeseler A, Jermiin LS. 2017. ModelFinder: fast model selection for accurate phylogenetic estimates. Nat Methods 14:587–589. doi:10.1038/nmeth.4285 28481363 PMC5453245

[68] Edwards DJ, Duchene S, Pope B, Holt KE. 2021. SNPPar: identifying convergent evolution and other homoplasies from microbial whole-genome alignments. Microb Genom 7:000694. doi:10.1099/mgen.0.000694 34874243 PMC8767352

[69] Pettersen EF, Goddard TD, Huang CC, Couch GS, Greenblatt DM, Meng EC, Ferrin TE. 2004. UCSF Chimera--a visualization system for exploratory research and analysis. J Comput Chem 25:1605–1612. doi:10.1002/jcc.20084 15264254

[70] Pettersen EF, Goddard TD, Huang CC, Meng EC, Couch GS, Croll TI, Morris JH, Ferrin TE. 2021. UCSF ChimeraX: structure visualization for researchers, educators, and developers. Protein Sci 30:70–82. doi:10.1002/pro.3943 32881101 PMC7737788

[71] Webb B, Sali A. 2021. Protein structure modeling with MODELLER, p 239–255. In Chen YW Yiu C-PB (ed), Structural genomics: general applications. Springer US, New York, NY.

[72] Wagner JM, Chan S, Evans TJ, Kahng S, Kim J, Arbing MA, Eisenberg D, Korotkov KV. 2016. Structures of EccB1 and EccD1 from the core complex of the mycobacterial ESX-1 type VII secretion system. BMC Struct Biol 16:5. doi:10.1186/s12900-016-0056-6 26922638 PMC4769845

[73] Evans R, O’Neill M, Pritzel A, Antropova N, Senior A, Green T, Žídek A, Bates R, Blackwell S, Yim J, Ronneberger O, Bodenstein S, Zielinski M, Bridgland A, Potapenko A, Cowie A, Tunyasuvunakool K, Jain R, Clancy E, Kohli P, Jumper J, Hassabis D. 2022. Protein complex prediction with Alphafold-Multimer. bioRxiv. doi:10.1101/2021.10.04.463034

[74] Conrad WH, Osman MM, Shanahan JK, Chu F, Takaki KK, Cameron J, Hopkinson-Woolley D, Brosch R, Ramakrishnan L. 2017. Mycobacterial ESX-1 secretion system mediates host cell lysis through bacterium contact-dependent gross membrane disruptions. Proc Natl Acad Sci U S A 114:1371–1376. doi:10.1073/pnas.1620133114 28119503 PMC5307465

[75] Schymkowitz J, Borg J, Stricher F, Nys R, Rousseau F, Serrano L. 2005. The FoldX web server: an online force field. Nucleic Acids Res 33:W382–8. doi:10.1093/nar/gki387 15980494 PMC1160148

[76] R Development Core Team. 2014. R: a language and environment for statistical computing, R foundation for statistical computing, http://www.R-project.org.

