# Supplementary figures and images for: Single nucleotide variation catalog from clinical isolates mapped on tertiary and quaternary structures of ESX-1-related proteins reveals critical regions as putative Mtb therapeutic targets
Source: Microbiol Spectr. 2024 Jun 14;12(8):e03816-23. doi: 10.1128/spectrum.03816-23 (PMC11302016; doi:10.1128/spectrum.03816-23)

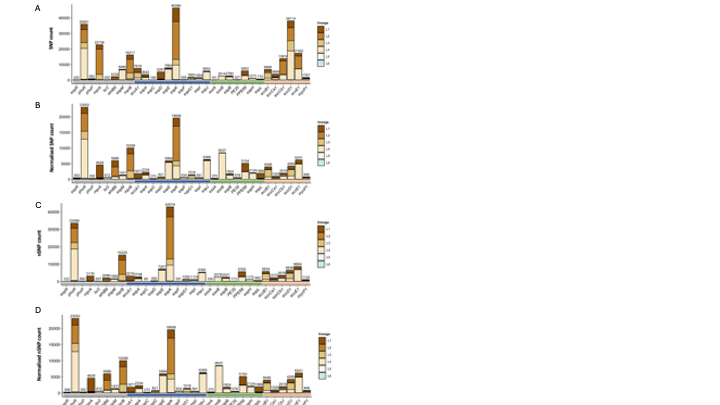

Supplement: Fig. S1 — Supplemental Figure 1. [file spectrum.03816-23-s0001.tiff]

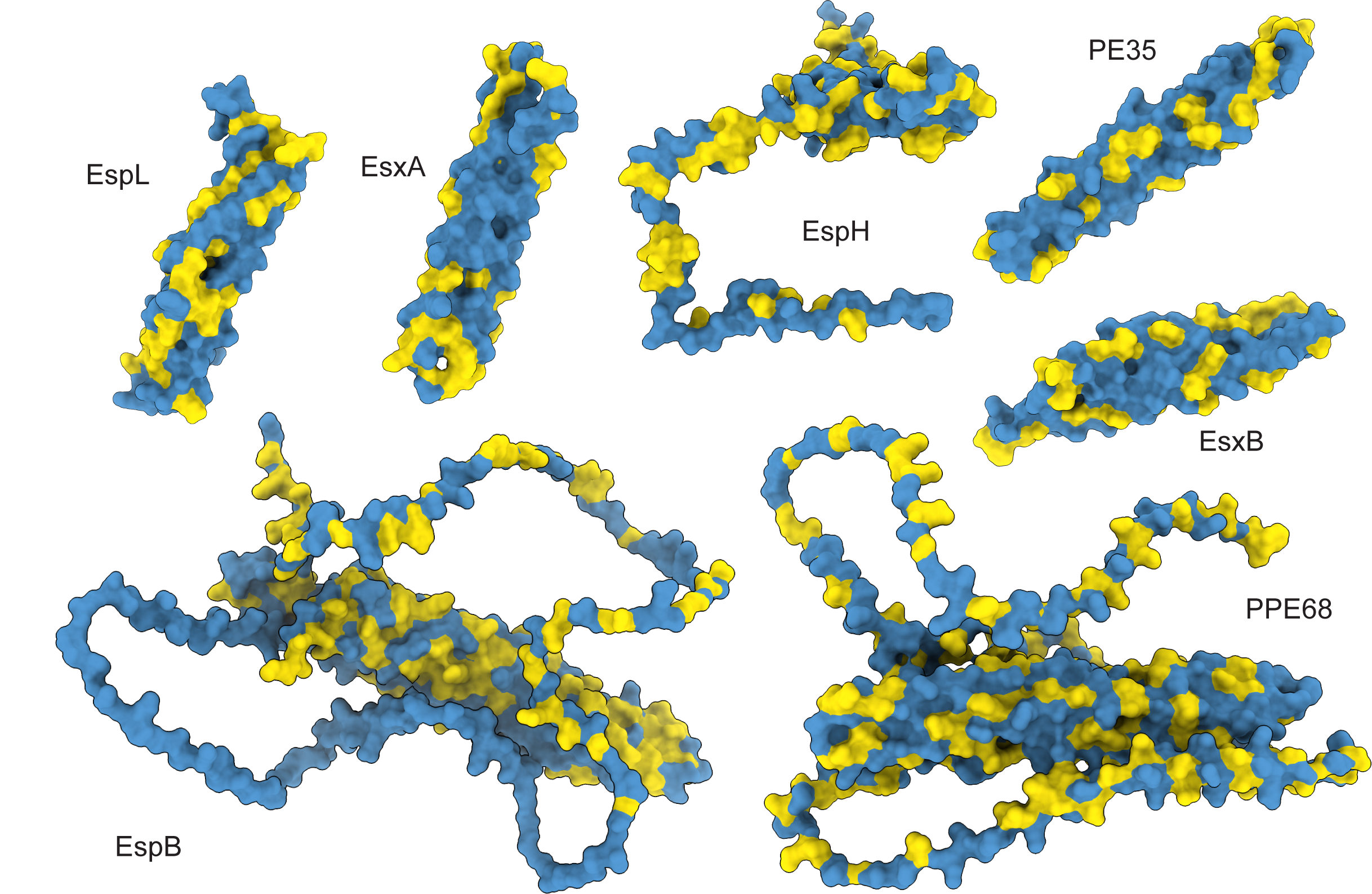

Supplement: Fig. S2 — Supplemental Figure 2. [file spectrum.03816-23-s0002.tif]

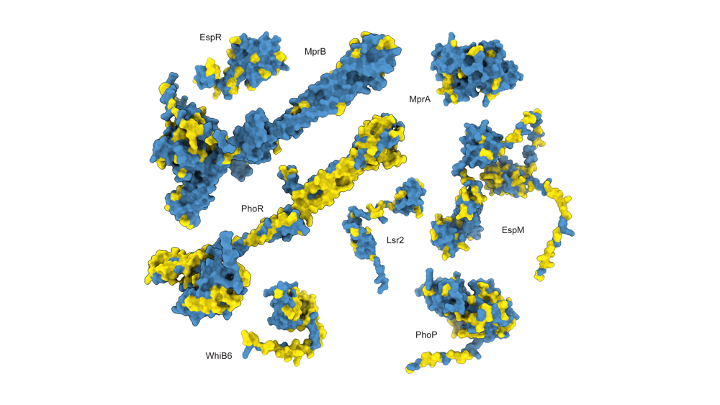

Supplement: Fig. S3 — Supplemental Figure 3. [file spectrum.03816-23-s0003.tiff]

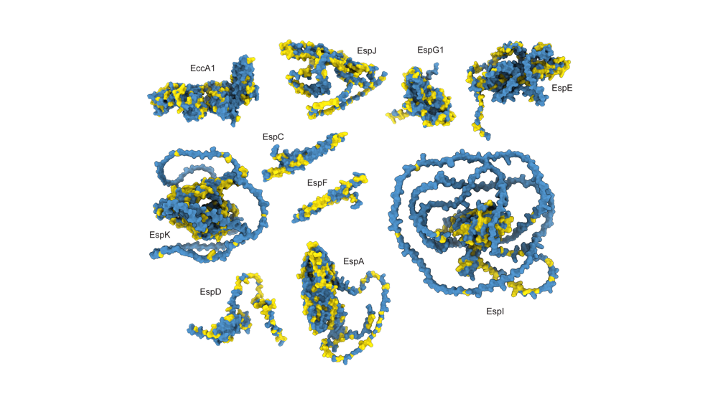

Supplement: Fig. S4 — Supplemental Figure 4. [file spectrum.03816-23-s0004.tiff]

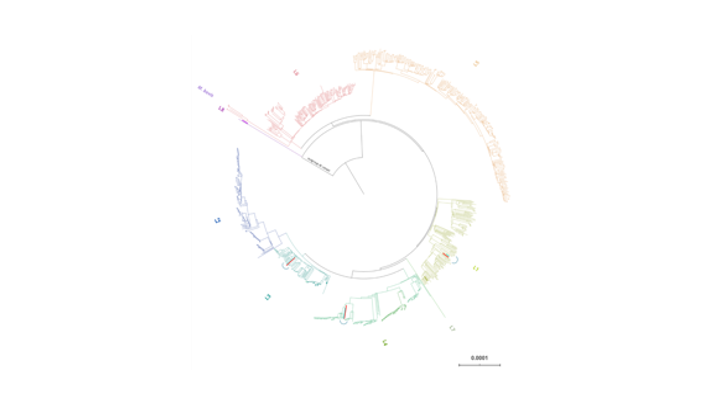

Supplement: Fig. S5 — Supplemental Figure 5. [file spectrum.03816-23-s0005.tiff]

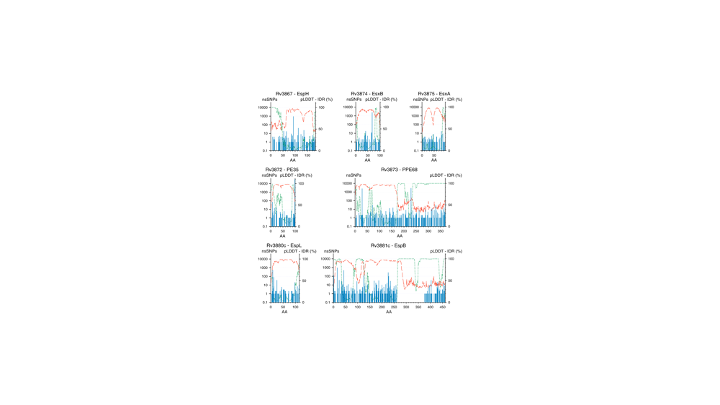

Supplement: Fig. S6 — Supplemental Figure 6. [file spectrum.03816-23-s0006.tiff]

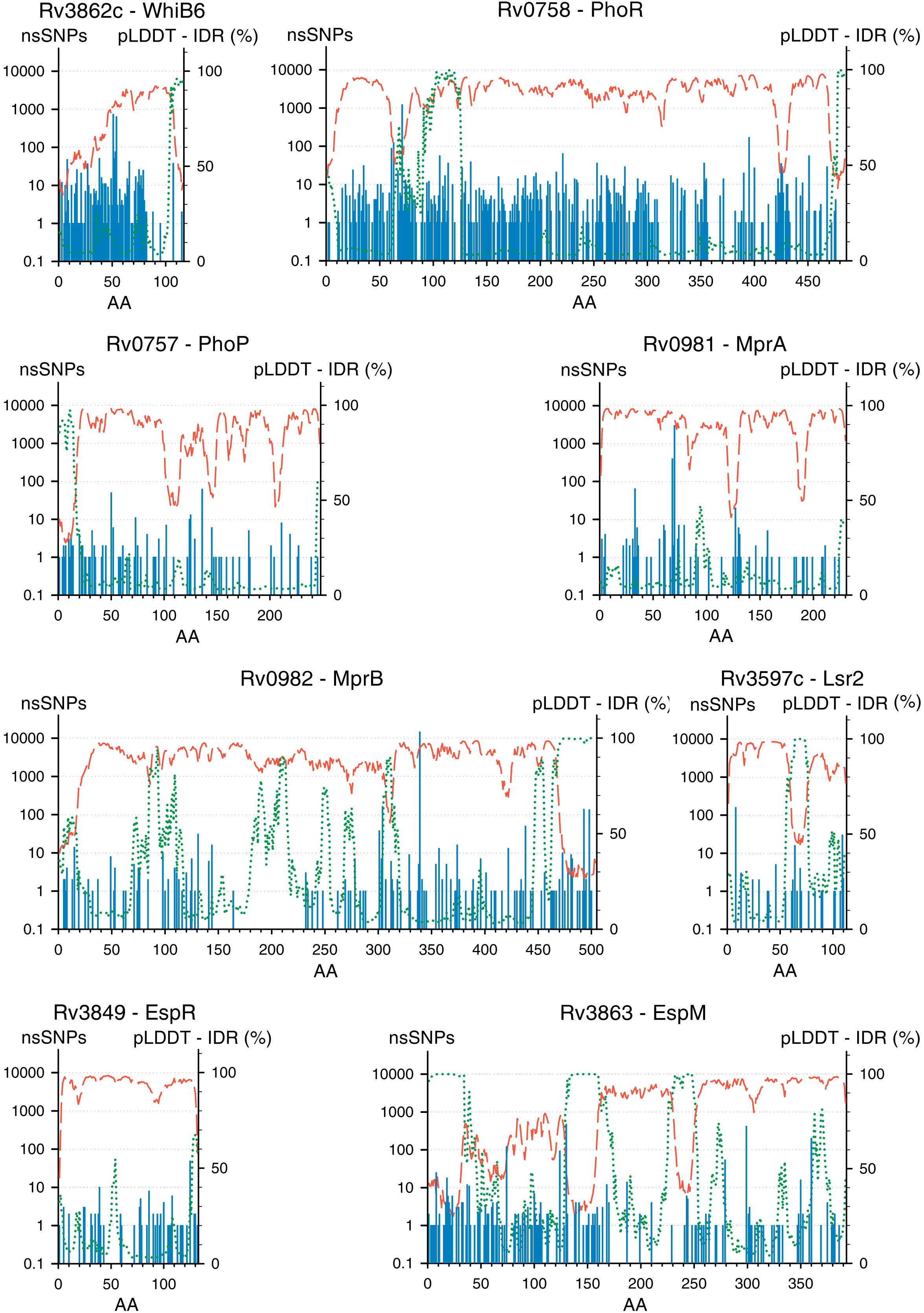

Supplement: Fig. S7 — Supplemental Figure 7. [file spectrum.03816-23-s0007.tif]

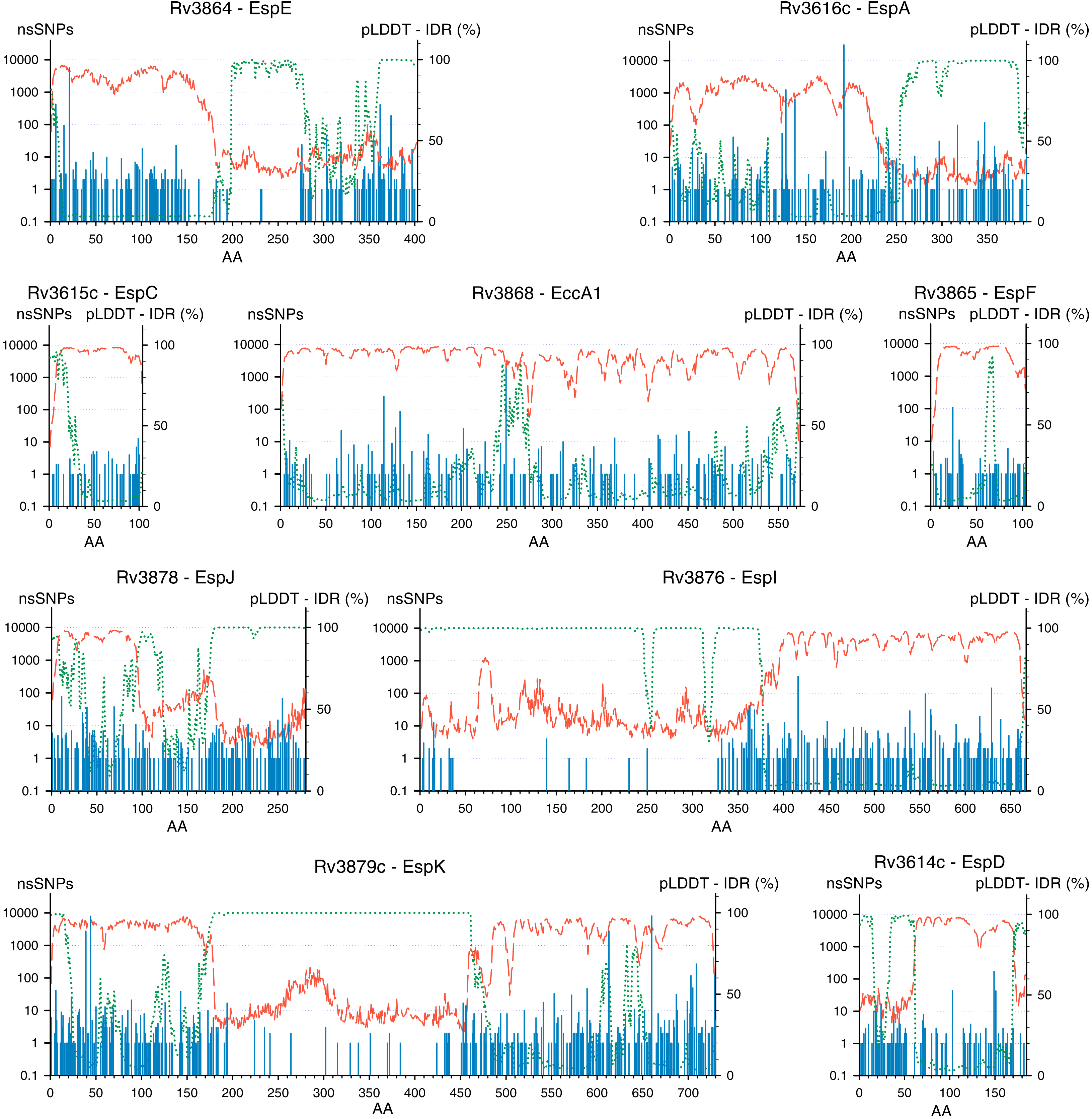

Supplement: Fig. S8 — Supplemental Figure 8. [file spectrum.03816-23-s0008.tif]

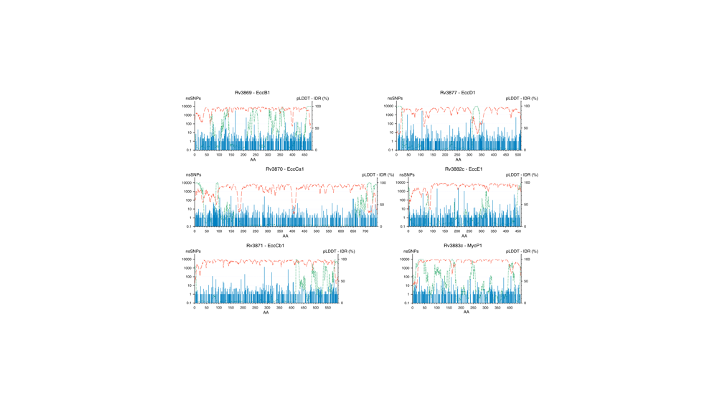

Supplement: Fig. S9 — Supplemental Figure 9. [file spectrum.03816-23-s0009.tiff]
